# Sodium-Glucose Cotransporter 2 (SGLT2) Inhibitors vs. Dipeptidyl Peptidase-4 (DPP4) Inhibitors for New-Onset Dementia: A Propensity Score-Matched Population-Based Study With Competing Risk Analysis

**DOI:** 10.3389/fcvm.2021.747620

**Published:** 2021-10-21

**Authors:** Jonathan V. Mui, Jiandong Zhou, Sharen Lee, Keith Sai Kit Leung, Teddy Tai Loy Lee, Oscar Hou In Chou, Shek Long Tsang, Abraham Ka Chung Wai, Tong Liu, Wing Tak Wong, Carlin Chang, Gary Tse, Qingpeng Zhang

**Affiliations:** ^1^Diabetes Research Unit, Cardiovascular Analytics Group, China-UK Collaboration, Hong Kong, China; ^2^School of Data Science, City University of Hong Kong, Hong Kong, China; ^3^Emergency Medicine Unit, Faculty of Medicine, The University of Hong Kong, Hong Kong, China; ^4^Tianjin Key Laboratory of Ionic-Molecular Function of Cardiovascular Disease, Department of Cardiology, Tianjin Institute of Cardiology, Second Hospital of Tianjin Medical University, Tianjin, China; ^5^State Key Laboratory of Agrobiotechnology (CUHK), School of Life Sciences, The Chinese University of Hong Kong, Hong Kong, China; ^6^Division of Neurology, Department of Medicine, Queen Mary Hospital, Hong Kong, China; ^7^Kent and Medway Medical School, Canterbury, United Kingdom

**Keywords:** SGLT2, SGLT2 (sodium-glucose cotransporter 2) inhibitor, DPP4, DPP4 inhibitor, dementia, cognitive dysfunction, Alzheimer's disease, Parkinson's disease

## Abstract

**Introduction:** The effects of sodium-glucose cotransporter 2 inhibitors (SGLT2I) and dipeptidyl peptidase-4 inhibitors (DPP4I) on new-onset cognitive dysfunction in type 2 diabetes mellitus remain unknown. This study aimed to evaluate the effects of the two novel antidiabetic agents on cognitive dysfunction by comparing the rates of dementia between SGLT2I and DPP4I users.

**Methods:** This was a population-based cohort study of type 2 diabetes mellitus patients treated with SGLT2I and DPP4I between January 1, 2015 and December 31, 2019 in Hong Kong. Exclusion criteria were <1-month exposure or exposure to both medication classes, or prior diagnosis of dementia or major neurological/psychiatric diseases. Primary outcomes were new-onset dementia, Alzheimer's, and Parkinson's. Secondary outcomes were all-cause, cardiovascular, and cerebrovascular mortality.

**Results:** A total of 13,276 SGLT2I and 36,544 DPP4I users (total *n* = 51,460; median age: 66.3 years old [interquartile range (IQR): 58–76], 55.65% men) were studied (follow-up: 472 [120–792] days). After 1:2 matching (SGLT2I: *n* = 13,283; DPP4I: *n* = 26,545), SGLT2I users had lower incidences of dementia (0.19 vs. 0.78%, *p* < 0.0001), Alzheimer's (0.01 vs. 0.1%, *p* = 0.0047), Parkinson's disease (0.02 vs. 0.14%, *p* = 0.0006), all-cause (5.48 vs. 12.69%, *p* < 0.0001), cerebrovascular (0.88 vs. 3.88%, *p* < 0.0001), and cardiovascular mortality (0.49 vs. 3.75%, *p* < 0.0001). Cox regression showed that SGLT2I use was associated with lower risks of dementia (hazard ratio [HR]: 0.41, 95% confidence interval [CI]: [0.27–0.61], *P* < 0.0001), Parkinson's (HR:0.28, 95% CI: [0.09–0.91], *P* = 0.0349), all-cause (HR:0.84, 95% CI: [0.77–0.91], *P* < 0.0001), cardiovascular (HR:0.64, 95% CI: [0.49–0.85], *P* = 0.0017), and cerebrovascular (HR:0.36, 95% CI: [0.3–0.43], *P* < 0.0001) mortality.

**Conclusions:** The use of SGLT2I is associated with lower risks of dementia, Parkinson's disease, and cerebrovascular mortality compared with DPP4I use after 1:2 ratio propensity score matching.

## Introduction

Type-2 diabetes mellitus is a complex multi-systemic disorder with wide-ranging complications affecting the retinal, cardiovascular, renal, and peripheral nervous systems (1–5). Increasingly, cognitive dysfunction is being recognized as a clinically important complication of type-2 diabetes (6). Diabetic patients are associated with a 1.5-fold increased risk of cognitive dysfunction, 1.9-fold increased risk of dementia, and 2.2-fold increased risk of stroke (7–9). While the underlying pathophysiology is still unclear, several mechanisms have been proposed including insulin resistance, hypoglycemia, hyperglycemia-induced cerebral microvascular and macrovascular dysfunction, as well as amyloid deposition (10, 11). It is highly likely that the cognitive dysfunction is multifactorial and caused by a combination of these mechanisms specific to the demographic and comorbidities of the patient.

Several studies have suggested that improved glycemic control, reduced HbA1c levels, and use of anti-diabetic medication are associated with a reduced risk of cognitive dysfunction (12–15). This has consequently raised the prospect of anti-diabetic agents reducing cognitive dysfunction in type 2 diabetes patients. Of interest are novel second-line anti-diabetic agents including sodium-glucose cotransporter 2 inhibitors (SGLT2I) and dipeptidyl peptidase-4 inhibitors. Multiple preclinical studies have suggested that DPP4I and SGLT2I improve cognition in animal models *via* a variety of mechanisms (16–20). However, few clinical studies have explored SGLT2I and DPP4I in their effects on cognitive dysfunction in diabetic patients. A randomized controlled trial in 2018 found no cognitive decline in SGLT2I and DPP4I users within 12 months while a case-control study in 2019 found that DPP4I and SGLT2I use are associated with a lower risk of dementia compared with other anti-diabetic agents (21, 22). Until recent times, no study has directly compared the risk of cognitive dysfunction and major neurocognitive disorders among SGLT2I and DPP4I users.

Therefore, the present study aimed to compare the incidence of dementia in SGLT2 users against DPP4I users in a Chinese population to evaluate the effects of the two novel antidiabetic agents on cognitive dysfunction.

## Methods

### Study Design and Population

**Figure 1:**
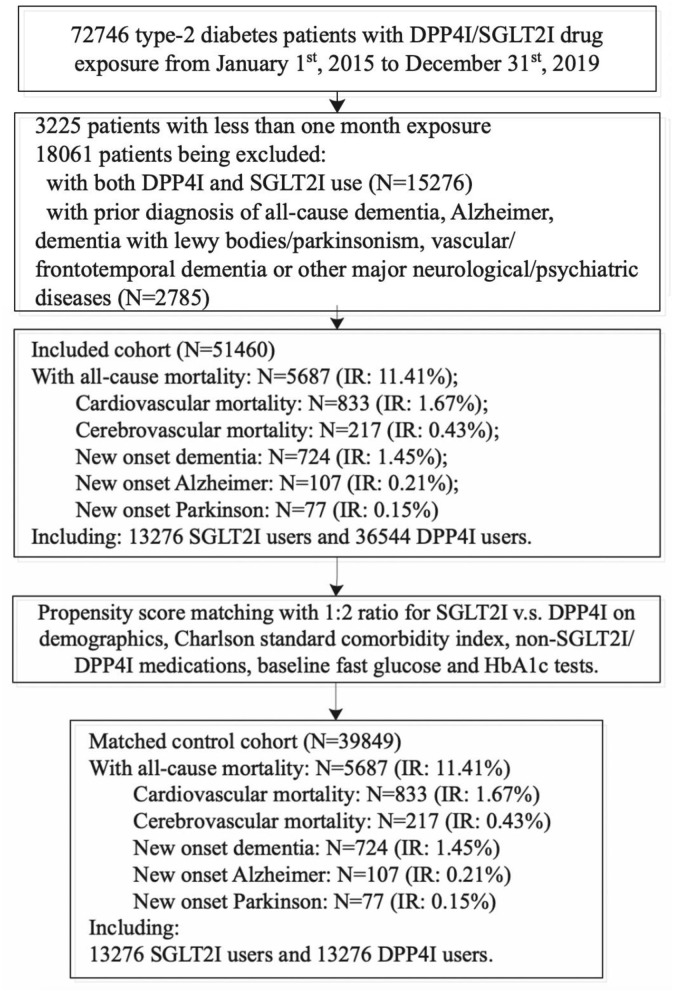
Procedures of data processing for the study cohort.

This was a retrospective, territory-wide cohort study of type-2 diabetes mellitus patients with SGLT2I/DPP4I use between January 1, 2015, and December 31, 2019 in Hong Kong (Figure 1). Patients during the aforementioned period were enrolled and followed up until December 31, 2019, or until death. Patients with <1 month SGLT2I/DPP4I exposure (*N* = 3,225), with both SGLT2I and DPP4I therapy (*N* = 15,276), or with a prior diagnosis of all-cause dementia, Alzheimer's disease, dementia with Lewy bodies, vascular dementia, frontotemporal dementia, or other major neurological/psychiatric diseases (*N* = 2,785) were excluded.

The patients were identified from the Clinical Data Analysis and Reporting System (CDARS), a city-wide database that centralizes patient information from individual local hospitals to establish comprehensive medical data, including clinical characteristics, disease diagnosis, laboratory results, and drug treatment details. The system has been previously used by both our team and other teams in Hong Kong (23–25). Clinical and biochemical data were extracted for the present study. The demographics of the patients include gender and age of initial drug use (baseline). Prior comorbidities were extracted based on standard *International Classification of Diseases Ninth Edition* (ICD-9) codes (Supplementary Table 1). The Charlson comorbidity index and neutrophil-to-lymphocyte ratio (NLR) were calculated. Mortality was recorded using the *International Classification of Diseases Tenth Edition* (ICD-10) coding. ICD-10 codes I00-I09, I11, I13, I20-I51 were used to identify cardiovascular mortality outcomes. ICD-10 codes I60-I69 identified cerebrovascular mortality. Medication histories and baseline laboratory examinations were extracted. Mortality data were obtained from the Hong Kong Death Registry, a population-based official government registry with the registered death records of all Hong Kong citizens linked to CDARS.

### Outcomes and Statistical Analysis

The primary outcomes were new-onset dementia, new-onset Alzheimer's disease, and new-onset Parkinson's disease. The secondary outcomes were all-cause mortality, cardiovascular mortality, and cerebrovascular mortality. Descriptive statistics were used to summarize baseline clinical and biochemical characteristics of patients with SGLT2I and DPP4I use. For baseline clinical characteristics, the continuous variables were presented as median (95% confidence interval [CI]/interquartile range [IQR]) and the categorical variables were presented as total number (percentage). Continuous variables were compared using the two-tailed Mann-Whitney U test, while the two-tailed Chi-square test with Yates' correction was used to test 2 × 2 contingency data. Propensity score matching with 1:2 ratio between SGLT2I and DPP4I users based on demographics, Charlson comorbidity index, prior comorbidities, non-SGLT2I/DPP4I medications, baseline fasting glucose, and HbA1c tests were performed using the nearest neighbor search strategy. Propensity score matching results between treatment-group (SGLT2I) vs. control-group (DPP4I) before and after matching are shown in Supplementary Figure 1. Propensity score matching adjustment approaches including propensity score stratification (26), propensity score matching with inverse probability weighting (27) and high-dimensional propensity score (28) were also performed.

Cox regression models were used to identify significant risk predictors for the study outcomes. Competing risk analysis models (cause-specific and sub-distribution) were considered. The hazard ratio (HR), 95% CI, and *P*-value were reported. Statistical significance is defined as *P* < 0.05. All statistical analyses were performed with R studio (Boston, MA, Version 1.1.456) and Python (Scotts Valley, CA, Version 3.6).

## Results

### Baseline Characteristics Before and After Propensity Score Matching

The study cohort included 13,276 SGLT2I users and 36,544 DPP4I users (total *n* = 51,460; median age: 66.3 years old [IQR: 58–76], 55.65% men). After a mean follow-up of 472 days (IQR: 120–792), 724 (1.45%) developed new-onset dementia, 107 (0.21%) developed new-onset Alzheimer's disease, 77 (0.15%) developed with new onset Parkinson's disease, and in total, 5,687 (11.41%) died from all-causes in which 833 (1.67%) died with cardiovascular causes and 217 (0.43%) died with cerebrovascular causes.

The baseline and clinical characteristics of DPP4I and SGLT2I users before and after 1:2 propensity score matching are shown in Table 1. Both before and after 1:2 propensity score matching, SGLT2I users had lower incidences of new-onset dementia (0.19 vs. 0.78%, *p* < 0.0001), new onset Alzheimer's disease (0.01 vs. 0.1%, *p* = 0.0047), new onset Parkinson's disease (0.02 vs. 0.14%, *p* = 0.0006), all-cause mortality (5.48 vs. 12.69%, *p* < 0.0001), cardiovascular mortality (0.49 vs. 3.75%, *p* < 0.0001), and cerebrovascular mortality (0.88 vs. 3.88%, *p* < 0.0001) compared with DPP4I users. The balancing comparisons of treated (SGLT2I) and controls (DPP4I) after 1:2 propensity matching with nearest neighbor search strategy are shown in Supplementary Table 2. None of the confounding characteristics remained significant after propensity matching.

**Table 1:** Baseline and clinical characteristics of patients with DPP4I vs. SGLT2I uses before and after propensity score matching (1:2).

**Characteristics**	**Before matching**			***P*-value**	**After matching**			***P*-value**
	**All (*N =* 39828) Median (IQR); N or Count(%)**	**SGLT2I users (*N =* 13276) Median (IQR); N or Count(%)**	**DPP4I (users *N =* 36554) Median (IQR); N or Count(%)**		**All (*N =* 49830) Median (IQR); N or Count(%)**	**SGLT2I users (*N =* 13283) Median (IQR); N or Count(%)**	**DPP4I users (*N =* 26545) Median (IQR); N or Count(%)**	
**Adverse events**								
All-cause mortality	5,687 (11.41%)	695 (5.23%)	4,992 (13.65%)	<0.0001^***^	5,687 (11.41%)	729 (5.48%)	3,371 (12.69%)	<0.0001^***^
Cardiovascular mortality	833 (1.67%)	108 (0.81%)	725 (1.98%)	<0.0001^***^	833 (1.67%)	66 (0.49%)	998 (3.75%)	<0.0001^***^
Cerebrovascular mortality	217 (0.43%)	18 (0.13%)	199 (0.54%)	<0.0001^***^	217 (0.43%)	117 (0.88%)	1,030 (3.88%)	<0.0001^***^
New onset dementia	724 (1.45%)	72 (0.54%)	652 (1.78%)	<0.0001^***^	724 (1.45%)	26 (0.19%)	208 (0.78%)	<0.0001^***^
New onset Alzheimer's	107 (0.21%)	12 (0.09%)	95 (0.25%)	0.0005^***^	107 (0.21%)	2 (0.01%)	27 (0.10%)	0.0047^**^
New onset Parkinson's	77 (0.15%)	10 (0.07%)	67 (0.18%)	0.0099^**^	77 (0.15%)	3 (0.02%)	39 (0.14%)	0.0006^***^
**Demographics**								
Male gender	27,734 (55.65%)	8,229 (61.98%)	19,505 (53.35%)	<0.0001^***^	27,734 (55.65%)	8,194 (61.68%)	15,714 (59.19%)	0.0175^*^
Female gender	22,096 (44.34%)	5,047 (38.01%)	17,049 (46.64%)	<0.0001^***^	22,096 (44.34%)	5,089 (38.31%)	10,831 (40.80%)	0.0017^**^
Baseline age, year	66.27 (58.08–75.59); *n =* 49,830	61.17 (53.89–68.42); *n =* 13,276	68.38 (59.92–77.97); *n =* 36,554	<0.0001^***^	66.27 (58.08–75.59); *n =* 49,830	61.18 (53.9–68.22); *n =* 13,283	62.08 (54.14–69.68); *n =* 26,545	<0.0001^***^
<40	1,161 (2.32%)	658 (4.95%)	503 (1.37%)	<0.0001^***^	1,161 (2.32%)	658 (4.95%)	1,304 (4.91%)	0.8837
[40, 50]	3,480 (6.98%)	1,553 (11.69%)	1,927 (5.27%)	<0.0001^***^	3,480 (6.98%)	1,552 (11.68%)	3,069 (11.56%)	0.7611
[50–60]	10,637 (21.34%)	3,831 (28.85%)	6,806 (18.61%)	<0.0001^***^	10,637 (21.34%)	3,829 (28.82%)	6,963 (26.23%)	<0.0001^***^
[60–70]	15,373 (30.85%)	4,495 (33.85%)	10,878 (29.75%)	<0.0001^***^	15,373 (30.85%)	4,579 (34.47%)	8,787 (33.10%)	0.0559
[70–80]	10,969 (22.01%)	1,979 (14.90%)	8,990 (24.59%)	<0.0001^***^	10,969 (22.01%)	1,965 (14.79%)	4,984 (18.77%)	<0.0001^***^
≥80	8,210 (16.47%)	760 (5.72%)	7,450 (20.38%)	<0.0001^***^	8,210 (16.47%)	700 (5.26%)	1,438 (5.41%)	0.5759
Charlson score	2.0 (1.0–3.0); *n =* 49,830	2.0 (1.0–3.0); *n =* 13,276	3.0 (2.0–4.0); *n =* 36,554	<0.0001^***^	2.0(1.0–3.0); *n =* 49,830	2.0 (1.0–3.0); *n =* 13,283	2.0 (1.0–3.0); *n =* 26,545	<0.0001^***^
NLR	2.39 (1.75–3.54); *n =* 19,776	2.17 (1.64–3.0); *n =* 5,560	2.5 (1.81–3.77); *n =* 14,216	<0.0001^***^	2.39 (1.75–3.54); *n =* 19,776	2.1 4(1.62–2.95); *n =* 5,597	2.13 (1.33–3.56); *n =* 10,176	0.9764
**Past comorbidities**								
Hypertension	11,993 (24.06%)	3,075 (23.16%)	8,918 (24.39%)	0.0262^*^	11,993 (24.06%)	3,036 (22.85%)	4,884 (18.39%)	<0.0001^***^
Heart failure	850 (1.70%)	208 (1.56%)	642 (1.75%)	0.167	850 (1.70%)	204 (1.53%)	253 (0.95%)	<0.0001^***^
Renal diseases	2,998 (6.01%)	193 (1.45%)	2,805 (7.67%)	<0.0001^***^	2,998 (6.01%)	178 (1.34%)	712 (2.68%)	<0.0001^***^
Liver diseases	351 (0.70%)	53 (0.39%)	298 (0.81%)	<0.0001^***^	351 (0.70%)	53 (0.39%)	114 (0.42%)	0.7193
Stroke/TIA	1,539 (3.08%)	390 (2.93%)	1,149 (3.14%)	0.2676	1,539 (3.08%)	385 (2.89%)	617 (2.32%)	0.0009^***^
Gastrointestinal bleeding	969 (1.94%)	204 (1.53%)	765 (2.09%)	0.0001^***^	969 (1.94%)	205 (1.54%)	313 (1.17%)	0.0033^**^
History of falls	3,405 (6.83%)	644 (4.85%)	2,761 (7.55%)	<0.0001^***^	3,405 (6.83%)	627 (4.72%)	1,134 (4.27%)	0.0529
Pneumonia and influenza	1,201 (2.41%)	156 (1.17%)	1,045 (2.85%)	<0.0001^***^	1,201 (2.41%)	143 (1.07%)	387 (1.45%)	0.0023^**^
Endocrine	1,047 (2.10%)	219 (1.64%)	828 (2.26%)	<0.0001^***^	1,047 (2.10%)	216 (1.62%)	416 (1.56%)	0.6931
Atrial fibrillation	2,139 (4.29%)	383 (2.88%)	1,756 (4.80%)	<0.0001^***^	2,139 (4.29%)	372 (2.80%)	1,323 (4.98%)	<0.0001^***^
Ischemic heart disease	5,355(10.74%)	1,811 (13.64%)	3,544 (9.69%)	<0.0001^***^	5,355(10.74%)	1,787 (13.45%)	2,339 (8.81%)	<0.0001^***^
Peripheral vascular disease	556 (1.11%)	86 (0.64%)	470 (1.28%)	<0.0001^***^	556 (1.11%)	82 (0.61%)	232 (0.87%)	0.0080^**^
Malignancy	1,380 (2.76%)	241 (1.81%)	1,139 (3.11%)	<0.0001^***^	1,380 (2.76%)	238 (1.79%)	278 (1.04%)	<0.0001^***^
Metastatic solid tumor	399 (0.80%)	42 (0.31%)	357 (0.97%)	<0.0001^***^	399 (0.80%)	42 (0.31%)	73 (0.27%)	0.5345
**Medications**								
SGLT2I vs. DPP4I	13,276 (26.64%)	13,276 (100.00%)	0 (0.00%)	<0.0001^***^	13,276 (26.64%)	13,283 (100.00%)	0 (0.00%)	<0.0001^***^
Beta blockers	1,547 (3.10%)	1,544 (11.63%)	3 (0.00%)	<0.0001^***^	1,547 (3.10%)	1,633 (12.29%)	2,557 (9.63%)	<0.0001^***^
Diuretics	1,378 (2.76%)	1,373 (10.34%)	5 (0.01%)	<0.0001^***^	1,378 (2.76%)	1,372 (10.32%)	699 (2.63%)	<0.0001^***^
Anticoagulants	49,566 (99.47%)	13,271 (99.96%)	36,295 (99.29%)	0.6437	49,566 (99.47%)	13,278 (99.96%)	26,535 (99.96%)	0.994
Antiplatelets	3,331 (6.68%)	3,320 (25.00%)	11 (0.03%)	<0.0001^***^	3,331 (6.68%)	3,408 (25.65%)	1,650 (6.21%)	<0.0001^***^
Antihypertensive drugs	1,007 (2.02%)	1,005 (7.57%)	2 (0.00%)	<0.0001^***^	1,007 (2.02%)	1,005 (7.56%)	2 (0.00%)	<0.0001^***^
Lipid–lowering drugs	7,394 (14.83%)	7,379 (55.58%)	15 (0.04%)	<0.0001^***^	7,394(14.83%)	7,467 (56.21%)	2,568 (9.67%)	<0.0001^***^
Statins and fibrates	7,226 (14.50%)	2,954 (22.25%)	4,272 (11.68%)	<0.0001^***^	7,226(14.50%)	2,932 (22.07%)	4,816 (18.14%)	<0.0001^***^
Non–steroidal anti–inflammatory drugs	3,152 (6.32%)	3,141(23.65%)	11 (0.03%)	<0.0001^***^	3,152 (6.32%)	3,229 (24.30%)	1,650 (6.21%)	<0.0001^***^
Other antidiabetic drugs	45,436 (91.18%)	11,341 (85.42%)	34,095 (93.27%)	<0.0001^***^	45,436(91.18%)	11,350 (85.44%)	22,735 (85.64%)	0.8878
**Complete blood counts**								
Mean corpuscular volume, fL	88.5 (85.0–91.7); *n =* 24,270	88.3 (84.9–91.3); *n =* 6,939	88.7 (85.0–91.9); *n =* 17,331	<0.0001^***^	88.5 (85.0–91.7); *n =* 24,270	88.3 (84.9–91.3); *n =* 6,967	89.6 (85.8–91.3); *n =* 12,055	<0.0001^***^
Basophil, × 10^∧^9/L	0.02 (0.0–0.05); *n =* 17,555	0.03 (0.0–0.06); *n =* 4,496	0.02 (0.0–0.05); *n =* 13,059	0.5161	0.02 (0.0–0.05); *n =* 17,555	0.02 (0.0–0.05); *n =* 4,538	0.03 (0.0–0.06); *n =* 9,599	<0.0001^***^
Eosinophil, × 10^∧^9/L	0.19 (0.1–0.3); *n =* 19,755	0.2 (0.1–0.3); *n =* 5,558	0.18 (0.1–0.3); *n =* 14,197	0.0061^**^	0.1 9 (0.1–0.3); *n =* 19,755	0.2 (0.1–0.3); *n =* 5,595	0.2 (0.1–0.22); *n =* 10,166	0.23
Lymphocyte, × 10^∧^9/L	1.9 (1.4–2.4); *n =* 19,776	2.06 (1.6–2.58); *n =* 5,560	1.81 (1.36–2.33); *n =* 14,216	<0.0001^***^	1.9 (1.4–2.4); *n =* 19,776	2.1 (1.63–2.56); *n =* 5,597	2.1 (1.46–2.6); *n =* 10,176	0.026^*^
Monocyte, × 10^∧^9/L	0.5 (0.38–0.6); *n =* 19,776	0.5 (0.4–0.6); *n =* 5,560	0.5 (0.37–0.6); *n =* 14,216	0.001^**^	0.5 (0.38–0.6); *n =* 19,776	0.5 (0.4–0.6); *n =* 5,597	0.5 (0.4–0.62); *n =* 10,176	<0.0001^***^
Neutrophil, × 10^∧^9/L	4.65 (3.67–6.08); *n =* 19,776	4.54 (3.61–5.86); *n =* 5,560	4.7 (3.69–6.18); *n =* 14,216	<0.0001^***^	4.65 (3.67–6.08); *n =* 19,776	4.5 (3.6–5.8); *n =* 5,597	4.4 (3.5–6.22); *n =* 10,176	0.307
White blood count, × 10^∧^9/L	7.48 (6.2–9.0); *n =* 24,278	7.5 (6.3–9.0); *n =* 6,946	7.43 (6.2–9.0); *n =* 17,332	0.0491^*^	7.48 (6.2–9.0); *n =* 24,278	7.5 (6.3–9.0); *n =* 6,974	7.71 (6.58–9.2); *n =* 12,054	<0.0001^***^
Mean cell haemoglobin, pg	29.9 (28.5–31.0); *n =* 24,270	29.8 (28.5–30.9); *n =* 6,939	29.9 (28.5–31.1); *n =* 17,331	0.0003^***^	29.9 (28.5–31.0); *n =* 24,270	29.8 (28.5–30.9); *n =* 6,967	30.2 (28.9–31.1); *n =* 12,055	<0.0001^***^
Platelet, × 10^∧^9/L	231.0 (190.0–277.0); *n =* 24,279	235.0 (197.0–280.0); *n =* 6,946	228.0 (188.0–276.0); *n =* 17,333	<0.0001^***^	231.0 (190.0–277.0); *n =* 24,279	236.0 (197.0–279.0); *n =* 6,974	238.0 (207.0–267.0); *n =* 12,054	0.0778
Red blood count, × 10^∧^12/L	4.46 (4.03–4.88); *n =* 24,270	4.7 (4.36–5.07); *n =* 6,939	4.36 (3.9–4.78); *n =* 17,331	<0.0001^***^	4.46 (4.03–4.88); *n =* 24,270	4.7 (4.35–5.07); *n =* 6,967	4.35 (4.17–4.85); *n =* 12,055	<0.0001^***^
**Liver and renal biochemical tests**								
K/Potassium, mmol/L	4.3 (4.0–4.6); *n =* 40,605	4.28 (4.0–4.51); *n =* 10,416	4.31 (4.01–4.7); *n =* 30,189	<0.0001^***^	4.3 (4.0–4.6); *n =* 40,605	4.3 (4.0–4.55); *n =* 10,429	4.3 (4.0–4.7); *n =* 20,235	<0.0001^***^
Urate, mmol/L	0.4 (0.32–0.48); *n =* 6,169	0.37 (0.3–0.44); *n =* 1,953	0.41 (0.34–0.49); *n =* 4,216	<0.0001^***^	0.4 (0.32–0.48); *n =* 6,169	0.37 (0.3–0.44); *n =* 1,943	0.4 (0.33–0.49); *n =* 2,394	<0.0001^***^
Albumin, g/L	42.0(39.4–44.0); *n =* 30, 323	43.0 (41.0–45.0); *n =* 8,761	41.8 (39.0–44.0); *n =* 21,562	<0.0001^***^	42.0 (39.4–44.0); *n =* 30,323	43.0 (40.9–45.0); *n =* 8,786	41.26 (38.1–44.0); *n =* 14,994	<0.0001^***^
Na/Sodium, mmol/L	139.8(138.0–141.0); *n =* 40, 626	139.9 (138.0–141.0); *n =* 10,420	139.78 (138.0–141.0); *n =* 30,206	0.0007^***^	139.8 (138.0–141.0); *n =* 40,626	139.86 (138.0–141.0); *n =* 10,433	139.0 (137.8–141.0); *n =* 20,241	<0.0001^***^
Urea, mmol/L	5.9 (4.7–7.7); *n =* 40,610	5.4 (4.5–6.59); *n =* 10,411	6.16 (4.8–8.26); *n =* 30,199	<0.0001^***^	5.9 (4.7–7.7); *n =* 40,610	5.4 (4.44–6.51); *n =* 10,424	5.7 (4.43–7.2); *n =* 20,241	<0.0001^***^
Protein, g/L	74.0 (70.2–77.1); *n =* 28,453	74.7 (71.1–78.0); *n =* 8,313	73.7 (70.0–77.0); *n =* 20,140	<0.0001^***^	74.0 (70.2–77.1); *n =* 28,453	74.6 (71.0–78.0); *n =* 8,340	73.4 (69.0–77.9); *n =* 14,208	<0.0001^***^
Creatinine, umol/L	82.0 (67.0–108.0); *n =* 40,731	76.0 (64.0–90.0); *n =* 10,428	86.0 (68.5–117.4); *n =* 30,303	<0.0001^***^	82.0 (67.0–108.0); *n =* 40,731	75.0 (64.0–89.2); *n =* 10,441	78.0 (65.0–99.0); *n =* 20,308	<0.0001^***^
Alkaline phosphatase, U/L	72.0 (59.0–88.0); *n =* 30,432	70.0 (58.0–85.1); *n =* 8,761	73.0 (60.0–89.0); *n =* 21,671	<0.0001^***^	72.0 (59.0–88.0); *n =* 30,432	70.0 (58.0–86.0); *n =* 8,786	71.0 (59.0–91.0); *n =* 15,083	<0.0001^***^
Aspartate transaminase, U/L	21.0 (16.0–29.0); *n =* 8,137	22.0 (17.0–30.25); *n =* 2,326	21.0 (15.0–28.0); *n =* 5,811	<0.0001^***^	21.0 (16.0–29.0); *n =* 8,137	21.1 (16.0–30.0); *n =* 2,382	19.0 (14.0–30.0); *n =* 5,846	<0.0001^***^
Alanine transaminase, U/L	22.0 (15.0–33.0); *n =* 24,264	26.0 (18.0–39.0); *n =* 6,993	20.0 (14.0–30.0); *n =* 17,271	<0.0001^***^	22.0 (15.0–33.0); *n =* 24,264	26.0 (18.0–39.0); *n =* 7,030	23.0 (17.0–32.0); *n =* 11,850	<0.0001^***^
Bilirubin, umol/L	10.0 (7.4–13.5); *n =* 30,260	10.2 (7.8–13.7); *n =* 8,741	10.0 (7.2–13.4); *n =* 21,519	<0.0001^***^	10.0 (7.4–13.5); *n =* 30,260	10.3 (7.8–13.9); *n =* 8,766	10.7 (8.0–15.0); *n =* 14,969	<0.0001^***^
**Glycemic and lipid profiles**								
Triglyceride, mmol/L	1.38 (0.97–2.0); *n =* 38,215	1.42 (1.0–2.09); *n =* 9,949	1.35 (0.96–1.98); *n =* 28,266	<0.0001^***^	1.38 (0.97–2.0); *n =* 38,215	1.44 (1.01–2.1); *n =* 9,973	1.4 (1.0–2.19); *n =* 18,675	0.0222^*^
Total cholesterol, mmol/L	4.08 (3.45–4.73); *n =* 38,246	4.14 (3.53–4.84); *n =* 9,956	4.05 (3.41–4.7); *n =* 28,290	<0.0001^***^	4.08 (3.45–4.73); *n =* 38,246	4.16 (3.53–4.87); *n =* 9,980	4.14 (3.41–4.94); *n =* 18,691	<0.0001^***^
Low–density lipoprotein (LDL), mmol/L	2.27 (1.83–2.79); *n =* 34,071	2.27 (1.83–2.85); *n =* 9,174	2.27 (1.84–2.76); *n =* 24,897	0.0879	2.27 (1.83–2.79); *n =* 34,071	2.28 (1.83–2.86); *n =* 9,206	2.36 (1.86–2.86); *n =* 16,683	0.0004^***^
High–density lipoprotein (LDL), mmol/L	1.14 (0.97–1.36); *n =* 34,635	1.13 (0.97–1.34); *n =* 9,344	1.14 (0.97–1.37); *n =* 25,291	0.0006^***^	1.14 (0.97–1.36); *n =* 34,635	1.13 (0.97–1.33); *n =* 9,375	1.12 (0.94–1.3); *n =* 17,006	<0.0001^***^
Fast glucose, mmol/L	7.9 (6.5–9.79); *n =* 34,961	8.0 (6.6–10.16); *n =* 8,745	7.89 (6.5–9.66); *n =* 26,216	<0.0001^***^	7.9 (6.5–9.79); *n =* 34,961	8.01 (6.6–10.24); *n =* 8,769	8.3 (6.71–10.9); *n =* 17,192	<0.0001^***^
HbA1C, g/dL	12.6 (10.5–14.0); *n =* 24,738	13.5 (11.8–14.6); *n =* 7,032	12.3 (10.1–13.7); *n =* 17,706	<0.0001^***^	12.6 (10.5–14.0); *n =* 24,738	13.5 (11.9–14.6); *n =* 7,059	13.0 (11.4–13.8); *n =* 12,259	<0.0001^***^

* *p ≤ 0.05*,

** *p ≤ 0.01*,

*** *p ≤ 0.001; SGLT2I, Sodium–glucose cotransporter−2 inhibitors; DPP4I, Dipeptidyl peptidase−4 inhibitors; NLR, neutrophil–to–lymphocyte ratio; TIA, transient ischemic attack*.

**Figure 2:**
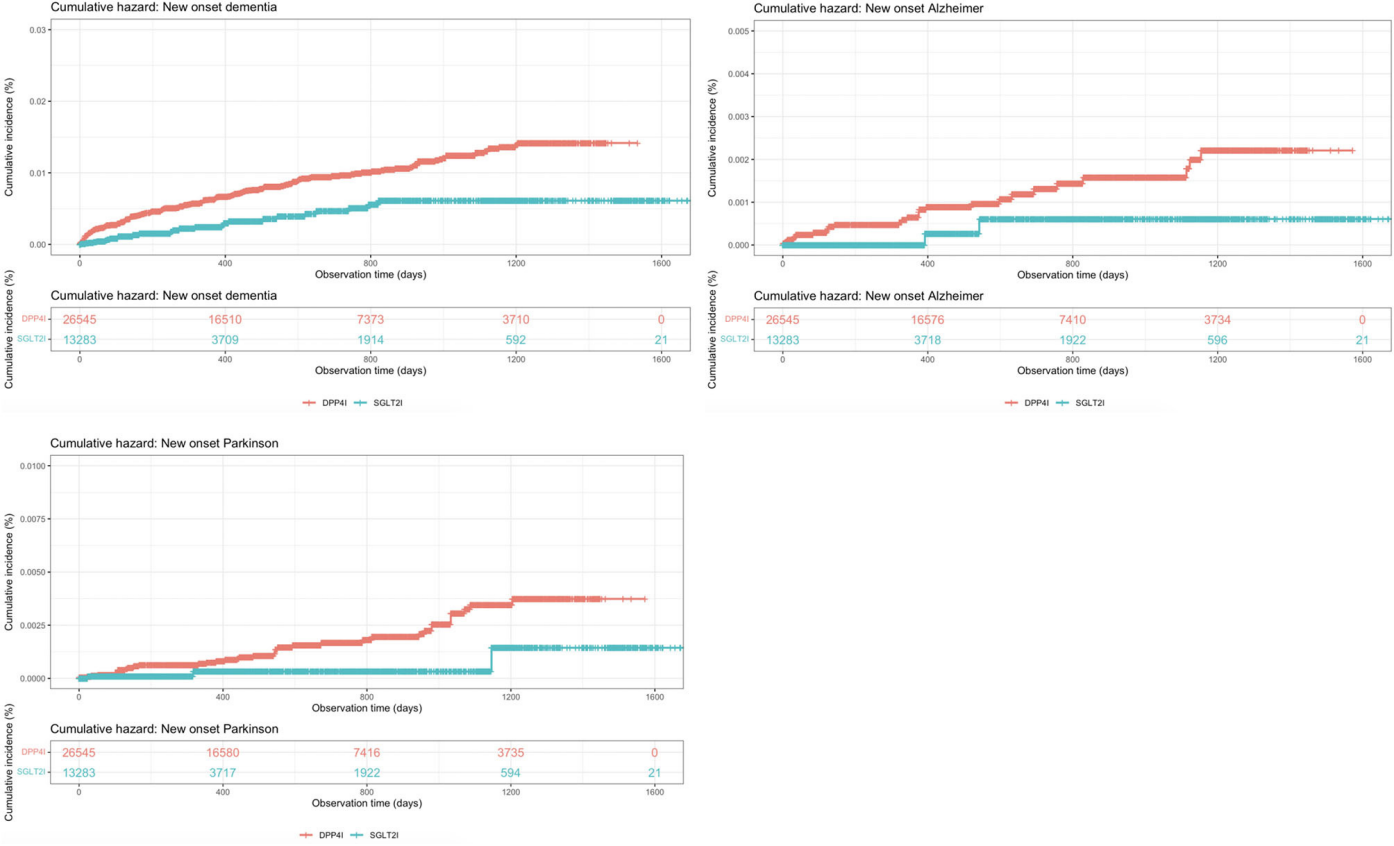
Cumulative incidence curves for new-onset cognitive dysfunctions stratified by the drug use of SGLT2I and DPP4I after propensity score matching (1:2).

**Figure 3:**
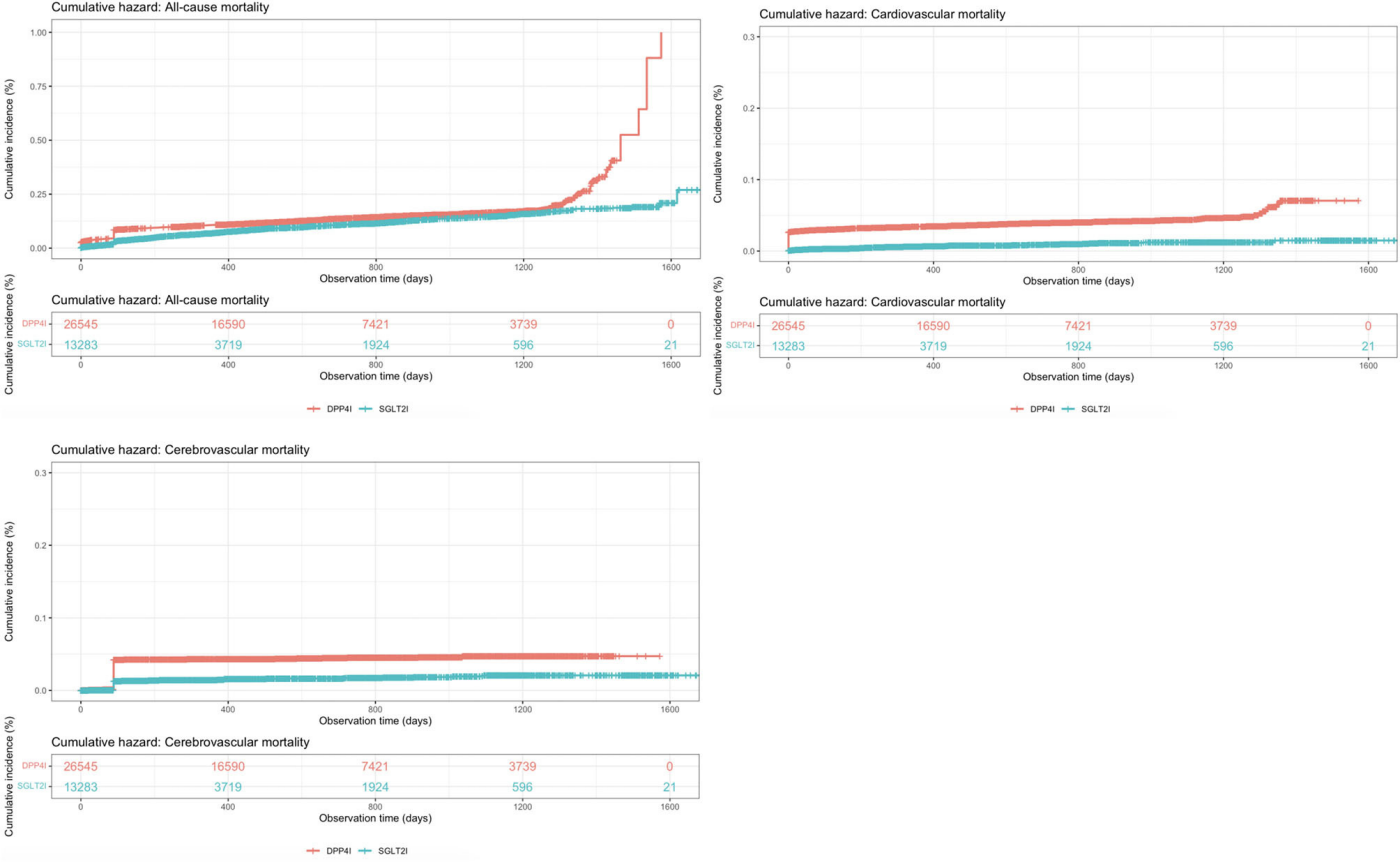
Cumulative incidence curves for mortality outcomes stratified by the drug use of SGLT2I and DPP4I after propensity score matching (1:2).

The baseline and clinical characteristics of patients with new-onset dementia, new-onset Alzheimer's, and new-onset Parkinson's before and after 1:2 propensity score matching are shown in Table 2. The cumulative incidence curves for new-onset cognitive dysfunction and mortality outcomes stratified by the drug use of SGLT2I and DPP4I after 1:2 propensity score matching are shown in Figures 2, 3, respectively.

**Table 2:** Baseline and clinical characteristics of patients with new–onset dementia, Alzheimer's, and Parkinson's before and after propensity score matching (1:2).

**Characteristics**	**Before matching**			***P*-value**	**After matching**			***P*-value**
	**New onset dementia (*N =* 724) Median (IQR); *N* or Count (%)**	**New onset Alzheimer's (*N =* 107) Median (IQR); *N* or Count (%)**	**New onset Parkinson's (*N =* 77) Median (IQR); *N* or Count (%)**		**New onset dementia (*N =* 234) Median (IQR); *N* or Count (%)**	**New onset Alzheimer's (*N =* 29) Median (IQR); *N* or Count (%)**	**New onset Parkinson's (*N =* 42) Median (IQR); *N* or Count (%)**	
**Demographics**
Male gender	131 (55.98%)	12 (41.37%)	30 (71.42%)	0.5571	298 (41.16%)	36 (33.64%)	45 (58.44%)	<0.0001^***^
Female gender	103 (44.01%)	17 (58.62%)	12 (28.57%)	0.4487	426 (58.83%)	71 (66.35%)	32 (41.55%)	<0.0001^***^
Baseline age, year	78.97 (68.6–84.2); *n =* 234	84.19 (77.67–87.79); *n =* 29	71.36 (63.64–77.66); *n =* 42	<0.0001^***^	81.72 (76.01–86.58); *n =* 724	83.68 (79.4–87.18); *n =* 107	77.29 (69.59–83.04); *n =* 77	<0.0001^***^
<40	0 (0.00%)	0 (0.00%)	0 (0.00%)	0.0012^**^	0 (0.00%)	0 (0.00%)	0 (0.00%)	<0.0001^***^
[40, 50]	3 (1.28%)	0 (0.00%)	0 (0.00%)	<0.0001^***^	1 (0.13%)	0 (0.00%)	0 (0.00%)	<0.0001^***^
[50–60]	11 (4.70%)	0 (0.00%)	7 (16.66%)	<0.0001^***^	13 (1.79%)	0 (0.00%)	5 (6.49%)	<0.0001^***^
[60–70]	55 (23.50%)	3 (10.34%)	13 (30.95%)	0.0199^*^	80 (11.04%)	4 (3.73%)	15 (19.48%)	<0.0001^***^
[70–80]	65 (27.77%)	8 (27.58%)	15 (35.71%)	0.0011^**^	202 (27.90%)	29 (27.10%)	27 (35.06%)	0.0030^**^
≥80	100 (42.73%)	18 (62.06%)	7 (16.66%)	<0.0001^***^	428 (59.11%)	74 (69.15%)	30 (38.96%)	<0.0001^***^
Charlson score	4.0 (3.0–4.0); *n =* 234	4.0 (3.0–4.0); *n =* 29	3.0 (2.0–3.0); *n =* 42	<0.0001^***^	4.0 (3.0–4.0); *n =* 724	4.0 (4.0–4.0); *n =* 107	3.0 (3.0–4.0); *n =* 77	<0.0001^***^
NLR	3.1 (1.92–4.97); *n =* 108	3.6 (1.87–7.99); *n =* 12	5.69 (2.18–14.2); *n =* 19	<0.0001^***^	3.08 (2.13–4.81); *n =* 362	3.11 (2.33–5.55); *n =* 44	3.23 (2.25–5.55); *n =* 33	<0.0001^***^
**Past comorbidities**								
Hypertension	76 (32.47%)	7 (24.13%)	13 (30.95%)	0.0002^***^	259 (35.77%)	26 (24.29%)	22 (28.57%)	<0.0001^***^
Heart failure	5 (2.13%)	1 (3.44%)	0 (0.00%)	0.2732	26 (3.59%)	3 (2.80%)	1 (1.29%)	0.0002^***^
Renal diseases	15 (6.41%)	2 (6.89%)	2 (4.76%)	<0.0001^***^	65 (8.97%)	7 (6.54%)	4 (5.19%)	0.0022^**^
Liver diseases	0 (0.00%)	0 (0.00%)	1 (2.38%)	0.6276	3 (0.41%)	0 (0.00%)	1 (1.29%)	0.4774
Stroke/TIA	11 (4.70%)	0 (0.00%)	0 (0.00%)	0.0631	39 (5.38%)	5 (4.67%)	1 (1.29%)	0.0008^***^
Gastrointestinal bleeding	9 (3.84%)	0 (0.00%)	2 (4.76%)	0.0021^**^	28 (3.86%)	4 (3.73%)	3 (3.89%)	0.0004^***^
History of falls	38 (16.23%)	4 (13.79%)	5 (11.90%)	<0.0001^***^	135 (18.64%)	20 (18.69%)	12 (15.58%)	<0.0001^***^
Pneumonia and influenza	16 (6.83%)	5 (17.24%)	2 (4.76%)	<0.0001^***^	51 (7.04%)	7 (6.54%)	4 (5.19%)	<0.0001^***^
Endocrine	6 (2.56%)	2 (6.89%)	3 (7.14%)	0.3606	22 (3.03%)	4 (3.73%)	1 (1.29%)	0.1102
Atrial fibrillation	12 (5.12%)	1 (3.44%)	0 (0.00%)	0.6375	48 (6.62%)	4 (3.73%)	0 (0.00%)	0.0041^**^
Ischemic heart disease	31 (13.24%)	4 (13.79%)	1 (2.38%)	0.2348	92 (12.70%)	13 (12.14%)	5 (6.49%)	0.1422
Peripheral vascular disease	1 (0.42%)	0 (0.00%)	0 (0.00%)	0.8017	12 (1.65%)	1 (0.93%)	2 (2.59%)	0.2298
Malignancy	8 (3.41%)	0 (0.00%)	0 (0.00%)	0.0115^*^	15 (2.07%)	1(0.93%)	1 (1.29%)	0.3124
Metastatic solid tumor	2 (0.85%)	0 (0.00%)	0 (0.00%)	0.3174	3 (0.41%)	0 (0.00%)	1 (1.29%)	0.3384
**Medications**								
SGLT2I vs. DPP4I	26 (11.11%)	2 (6.89%)	3 (7.14%)	<0.0001^***^	72 (9.94%)	12 (11.21%)	10 (12.98%)	<0.0001^***^
Beta blockers	1 (0.42%)	0 (0.00%)	0 (0.00%)	<0.0001^***^	1 (0.13%)	0 (0.00%)	0 (0.00%)	<0.0001^***^
Diuretics	0 (0.00%)	0 (0.00%)	0 (0.00%)	0.0008^***^	0 (0.00%)	0 (0.00%)	0 (0.00%)	<0.0001^***^
Anticoagulants	234 (100.00%)	29 (100.00%)	42 (100.00%)	0.9663	717 (99.03%)	106 (99.06%)	76 (98.70%)	0.954
Antiplatelets	1 (0.42%)	0 (0.00%)	0 (0.00%)	<0.0001^***^	2 (0.27%)	0 (0.00%)	0 (0.00%)	<0.0001^***^
Antihypertensive drugs	2 (0.85%)	0 (0.00%)	0 (0.00%)	0.1623	2 (0.27%)	0 (0.00%)	0 (0.00%)	0.0014^**^
Lipid–lowering drugs	4 (1.70%)	1 (3.44%)	0 (0.00%)	<0.0001^***^	5 (0.69%)	1 (0.93%)	0 (0.00%)	<0.0001^***^
Statins and fibrates	26 (11.11%)	3 (10.34%)	10 (23.80%)	0.0076^**^	84 (11.60%)	10 (9.34%)	11 (14.28%)	0.0575
Non–steroidal anti–inflammatory drugs	1 (0.42%)	0 (0.00%)	0 (0.00%)	<0.0001^***^	2 (0.27%)	0 (0.00%)	0 (0.00%)	<0.0001^***^
Other antidiabetic drugs	210 (89.74%)	28 (96.55%)	37 (88.09%)	0.6502	685 (94.61%)	100 (93.45%)	72 (93.50%)	0.502
**Complete blood counts**								
Mean corpuscular volume, fL	90.0 (87.15–92.95); *n =* 127	88.7 (87.3–94.1); *n =* 15	89.3 (86.0–90.0); *n =* 25	0.0006^***^	89.0 (85.65–92.3); *n =* 427	88.4 (85.95–90.9); *n =* 55	88.75 (84.3–91.4); *n =* 44	0.023^*^
Basophil, × 10^∧^9/L	0.02 (0.0–0.04); *n =* 96	0.04 (0.0–0.07); *n =* 12	0.02 (0.0–0.05); *n =* 16	0.0242^*^	0.02 (0.0–0.04); *n =* 322	0.03 (0.0–0.05); *n =* 41	0.03 (0.02–0.04); *n =* 28	0.013^*^
Eosinophil, × 10^∧^9/L	0.15 (0.08–0.29); *n =* 108	0.19 (0.0–0.24); *n =* 12	0.1 (0.05–0.1); *n =* 19	0.0385^*^	0.17 (0.1–0.3); *n =* 361	0.2 (0.1–0.27); *n =* 44	0.13 (0.09–0.2); *n =* 33	0.0379^*^
Lymphocyte, × 10^∧^9/L	1.53 (1.04–1.98); *n =* 108	1.28 (0.9–1.93); *n =* 12	1.37 (0.84–1.5); *n =* 19	<0.0001^***^	1.56 (1.1–2.1); *n =* 362	1.45 (1.06–1.98); *n =* 44	1.4 (0.99–1.9); *n =* 33	<0.0001^***^
Monocyte, × 10^∧^9/L	0.47 (0.36–0.6); *n =* 108	0.5 (0.38–0.8); *n =* 12	0.5 (0.26–0.65); *n =* 19	0.1121	0.5 (0.36–0.6); *n =* 362	0.5 (0.37–0.6); *n =* 44	0.5 (0.33–0.7); *n =* 33	0.1745
Neutrophil, × 10^∧^9/L	4.61 (3.6–6.6); *n =* 108	5.15 (3.4–7.75); *n =* 12	7.32 (3.4–11.79); *n =* 19	0.6069	4.8 (3.78–6.59); *n =* 362	5.11 (4.04–6.48); *n =* 44	5.2 (3.44–7.32); *n =* 33	0.0402^*^
White blood count, × 10^∧^9/L	7.31 (5.85–9.5); *n =* 127	7.3 (5.38–10.51); *n =* 15	8.5 (5.1–9.42); *n =* 25	0.4192	7.6 (6.2–9.1); *n =* 427	7.49 (6.41–8.96); *n =* 55	7.28 (5.62–9.28); *n =* 44	0.5659
Mean cell haemoglobin, pg	30.2 (29.1–31.6); *n =* 127	29.5 (29.0–31.2); *n =* 15	29.9 (29.3–30.9); *n =* 25	0.0144^*^	30.0 (28.6–31.2); *n =* 427	30.0 (28.6–31.0); *n =* 55	29.85 (28.85–31.25); *n =* 44	0.1277
Platelet, × 10^∧^9/L	220.0 (175.5–274.5); *n =* 127	190.0 (153.5–223.5); *n =* 15	239.0 (222.0–275.0); *n =* 25	0.0018^**^	223.0 (184.0–271.0); *n =* 427	217.0 (183.0–265.0); *n =* 55	234.0 (200.5–277.0); *n =* 44	0.0208^*^
Red blood count, × 10^∧^12/L	4.07 (3.56–4.44); *n =* 127	4.16 (3.38–4.44); *n =* 15	4.29 (3.96–4.77); *n =* 25	<0.0001^***^	4.08 (3.63–4.51); *n =* 427	4.25 (3.73–4.52); *n =* 55	4.16 (3.82–4.5); *n =* 44	<0.0001^***^
**Liver and renal biochemical tests**								
K/Potassium, mmol/L	4.3 (4.0–4.7); *n =* 190	4.3 (3.9–4.5); *n =* 24	4.3 (4.01–4.7); *n =* 37	0.8572	4.32 (4.0–4.7); *n =* 621	4.2 (4.1–4.5); *n =* 87	4.4 (4.2–4.7); *n =* 65	0.3277
Urate, mmol/L	0.35 (0.29–0.48); *n =* 31	0.36 (0.29–0.39); *n =* 4	0.35 (0.32–0.36); *n =* 10	0.3769	0.4 (0.34–0.48); *n =* 82	0.39 (0.34–0.43); *n =* 9	0.33 (0.31–0.38); *n =* 14	0.5403
Albumin, g/L	40.0 (37.0–42.45); *n =* 146	38.2 (36.5–42.37); *n =* 19	42.0 (38.5–45.1); *n =* 32	<0.0001^***^	39.78 (37.0–42.21); *n =* 494	40.95 (37.0–43.0); *n =* 68	40.0 (36.85–42.25); *n =* 48	<0.0001^***^
Na/Sodium, mmol/L	139.0 (137.0–142.0); *n =* 190	140.1 (137.5–142.5); *n =* 24	138.0 (137.0–140.7); *n =* 37	0.4646	139.4 (137.0–141.4); *n =* 621	140.0 (137.0–142.25); *n =* 87	138.0 (136.5–141.0); *n =* 65	0.1776
Urea, mmol/L	6.5 (5.2–8.8); *n =* 190	6.01 (4.88–9.71); *n =* 24	6.0 (4.4–7.28); *n =* 37	<0.0001^***^	6.9 (5.2–9.64); *n =* 619	6.82 (5.12–9.22); *n =* 87	6.6 (4.92–9.13); *n =* 65	<0.0001^***^
Protein, g/L	72.0 (67.9–76.0); *n =* 137	71.84 (66.5–79.0); *n =* 16	72.0 (69.5–77.0); *n =* 29	0.0005^***^	72.35 (68.45–77.0); *n =* 466	72.5 (68.25–77.2); *n =* 63	71.1 (68.25–75.8); *n =* 43	<0.0001^***^
Creatinine, umol/L	97.5 (73.6–134.0); *n =* 190	104.0 (71.5–125.0); *n =* 24	90.0 (67.5–126.0); *n =* 37	<0.0001^***^	99.0 (77.3–132.8); *n =* 621	93.9 (71.0–121.0); *n =* 87	94.7 (71.0–126.0); *n =* 65	<0.0001^***^
Alkaline phosphatase, U/L	76.0 (64.0–90.0); *n =* 146	70.0 (63.0–85.5); *n =* 19	69.85 (60.0–95.0); *n =* 32	0.0012^**^	76.0 (63.0–93.5); *n =* 495	72.35 (64.1–86.0); *n =* 68	71.0 (59.5–92.0); *n =* 48	<0.0001^***^
Aspartate transaminase, U/L	17.0 (13.0–23.0); *n =* 42	14.0 (13.0–17.95); *n =* 4	21.0 (16.5–25.0); *n =* 8	0.0196^*^	18.0 (13.0–25.0); *n =* 127	18.45 (13.0–25.5); *n =* 16	19.0 (13.5–25.0); *n =* 12	0.0003^***^
Alanine transaminase, U/L	16.0 (12.0–21.4); *n =* 120	14.0 (14.0–19.0); *n =* 17	25.0 (18.0–49.0); *n =* 25	<0.0001^***^	17.0 (12.0–24.5); *n =* 379	16.0 (13.0–20.45); *n =* 56	18.0 (11.0–34.0); *n =* 39	<0.0001^***^
Bilirubin, umol/L	10.0 (6.8–13.9); *n =* 146	9.9 (6.9–11.1); *n =* 19	11.9 (8.55–15.0); *n =* 32	0.0409^*^	9.0 (6.6–12.7); *n =* 493	9.0 (6.52–11.35); *n =* 68	9.7 (6.0–13.55); *n =* 48	<0.0001^***^
**Glycemic and lipid profiles**								
Triglyceride, mmol/L	1.22 (0.86–1.66); *n =* 165	1.22 (0.85–1.61); *n =* 19	1.25 (0.94–1.98); *n =* 32	<0.0001^***^	1.29 (0.95–1.78); *n =* 522	1.1 (0.88–1.58); *n =* 70	1.15 (0.83–1.84); *n =* 60	0.0077^**^
Total cholesterol, mmol/L	3.85 (3.34–4.8); *n =* 166	3.8 (3.49–4.47); *n =* 19	3.98 (2.58–4.61); *n =* 33	0.069	3.9 (3.27–4.6); *n =* 523	3.78 (2.91–4.31); *n =* 70	3.91 (3.1–4.51); *n =* 61	0.0009^***^
Low–density lipoprotein (LDL), mmol/L	2.21 (1.79–2.7); *n =* 144	2.17 (1.61–2.35); *n =* 17	1.98 (1.75–2.76); *n =* 25	0.4206	2.15 (1.72–2.72); *n =* 449	1.87 (1.59–2.42); *n =* 61	2.14 (1.75–2.71); *n =* 49	0.0068^**^
High–density lipoprotein (LDL), mmol/L	1.21 (1.03–1.5); *n =* 144	1.2 (1.01–1.53); *n =* 17	1.2 (1.08–1.4); *n =* 26	<0.0001^***^	1.17 (0.99–1.45); *n =* 454	1.2 (1.0–1.5); *n =* 61	1.18 (1.02–1.53); *n =* 50	0.0283^*^
Fast glucose, mmol/L	7.54 (6.0–10.87); *n =* 170	7.13 (6.1–9.73); *n =* 20	8.68 (6.95–10.52); *n =* 32	0.0667	7.99 (6.1–10.36); *n =* 522	7.26 (5.9–9.52); *n =* 64	8.14 (6.5–10.22); *n =* 54	0.9507
HbA1C, g/dL	11.9 (10.5–13.3); *n =* 131	12.2 (10.45–13.25); *n =* 15	12.45 (11.3–13.6); *n =* 26	<0.0001^***^	11.6 (9.7–13.0); *n =* 436	12.2 (10.2–13.5); *n =* 57	11.4 (10.4–12.94); *n =* 45	<0.0001^***^

* *p ≤ 0.05*,

** *p ≤ 0.01*,

*** *p ≤ 0.001; SGLT2I, Sodium–glucose cotransporter−2 inhibitors; DPP4I, Dipeptidyl peptidase−4 inhibitors; NLR, neutrophil–to–lymphocyte ratio; TIA, transient ischemic attack*.

### Univariate Cox Regression Analyses

The univariate Cox analyses of significant risk factors for new-onset dementia, Alzheimer's, and Parkinson's disease are shown in Supplementary Table 3 while the univariate Cox analyses of significant risk factors for all-cause, cardiovascular, and cerebrovascular mortality are shown in Supplementary Table 4. Compared with DPP4I, SGLT2I use demonstrated significant protective effects against new onset dementia (HR:0.41, 95% CI: [0.27, 0.61], *P* <0.0001) and new onset Parkinson's disease (HR:0.28, 95% CI: [0.09, 0.91], *P* = 0.0349), but not new onset Alzheimer's disease (HR:0.25, 95% CI: [0.06, 1.04], *P* = 0.0569). SGLT2 use was also associated with significantly lower incidence of all-cause mortality (HR:0.84, 95% CI: [0.77, 0.91], *P* < 0.0001), cardiovascular mortality (HR:0.64, 95% CI: [0.49, 0.85], *P* = 0.0017), and cerebrovascular mortality (HR:0.36, 95% CI: [0.30, 0.43], *P* < 0.0001).

### Sensitivity Analysis With Competing Risk Consideration

Competing for risk analyses using cause-specific and subdistribution hazard models were conducted on the matched cohorts as presented in Table 3. Both models confirmed the findings from the univariate Cox analyses that SGLT2I use is associated with lower incidence of new-onset dementia, new-onset Parkinson's, all-cause mortality, cardiovascular mortality, and cerebrovascular mortality, but not new-onset Alzheimer's disease compared with DPP4I use. In addition, sensitivity analyses were further conducted using Cox proportional hazard model on the matched cohorts with 1-year lag time, as presented in Supplementary Table 5.

**Table 3:** HRs (and 95% CIs) of SGLT2I vs. DPP4I from cause–specific and subdistribution hazard models for cognitive dysfunction and mortality risks after 1:2 propensity score matching.

**Model**	**Adverse outcomes**	**SGLT2I vs. DPP4I**
		**(After 1:2 matching)**
		**HR [95% CI]; *P*-value**
Cause–specific model	New onset Parkinson's	0.28 [0.09–0.91]; 0.0347^*^
	New onset Alzheimer's	0.25 [0.06–1.04]; 0.0567.
	New onset dementia	0.43 [0.28–0.66]; 0.0002^***^
	Cerebrovascular mortality	0.55 [0.29–0.73]; <0.0001^***^
	Cardiovascular mortality	0.45 [0.31–0.59]; <0.0001^***^
	All–cause mortality	0.54 [1.45–0.78]; <0.0001^***^
Sub–distribution model	New onset Parkinson's	0.32 [0.12–0.89]; 0.0209^*^
	New onset Alzheimer's	0.29 [0.09–1.05]; 0.0502.
	New onset dementia	0.48 [0.31–0.72]; 0.0001^***^
	Cerebrovascular mortality	0.39 [0.22–0.59]; <0.0001^***^
	Cardiovascular mortality	0.55 [0.23–0.71]; <0.0001^***^
	All–cause mortality	0.54 [0.38–0.69]; <0.0001^***^

* *p ≤ 0.05*,

** *p ≤ 0.01*,

*** *p ≤ 0.001; SGLT2I, Sodium–glucose cotransporter−2 inhibitors; DPP4I, Dipeptidyl peptidase−4 inhibitors; HR, hazard ratio; CI, confidence interval*.

Finally, different propensity score matching adjustment approaches were performed as presented in Table 4. Again, the three approaches confirmed the findings from the univariate Cox analyses that SGLT2I users have a lower risk of new-onset dementia, new-onset Parkinson's, all-cause mortality, cardiovascular mortality, and cerebrovascular mortality, but not new-onset Alzheimer's disease compared with DPP4I users.

**Table 4:** Risk of incident adverse cognitive dysfunction events, and mortality outcomes in matched cohorts associated with the treatment of SGLT2I vs. DPP4I using different matching approaches.

**Outcome**	**HR after PS stratification**	**HR after HDPS matching**	**HR after PS IPTW**
	**[95% CI]; *P*-value**	**[95% CI]; *P*-value**	**[95% CI]; *P*-value**
New onset Parkinson's	0.3 [0.13–0.9]; 0.0343^*^	0.25 [0.08–0.92]; 0.0357^*^	0.31 [0.09–0.87]; 0.0357^*^
New onset Alzheimer's	0.26 [0.08–1.02]; 0.0564.	0.28 [0.05–1.04]; 0.0557.	0.21 [0.01–1.02]; 0.0553.
New onset dementia	0.41 [0.29–0.75]; 0.0003^***^	0.46 [0.31–0.79]; 0.0014^**^	0.46 [0.3–0.75]; 0.0007^***^
Cerebrovascular mortality	0.42 [0.3–0.83]; <0.0001^***^	0.43 [0.29–0.8]; <0.0001^***^	0.47 [0.26–0.84]; <0.0001^***^
Cardiovascular mortality	0.61 [0.32–0.9]; <0.0001^***^	0.57 [0.32–0.89]; <0.0001^***^	0.43 [0.29–0.87]; <0.0001^***^
All–cause mortality	0.78 [0.62–0.84]; <0.0001^***^	0.72 [0.63–0.89]; <0.0001^***^	0.78 [0.62x−0.87]; <0.0001^***^

* *p ≤ 0.05*,

** *p ≤ 0.01*,

*** *p ≤ 0.001; SGLT2I, Sodium–glucose cotransporter−2 inhibitors; DPP4I, Dipeptidyl peptidase−4 inhibitors; HR, hazard ratio; CI, confidence interval; PS, propensity score; HDPS, high dimensional propensity score; IPTW, inverse probability of treatment weighting*.

### Subgroup Analysis

A subgroup analysis was performed on SGLT2I and DPP4I users with concurrent type-2 diabetes and cardiovascular disease (defined as heart failure, myocardial infarction, ischemic heart disease, peripheral vascular disease, atrial fibrillation, or cardiovascular medication use) (Table 5). Patients with new-onset cardiovascular disease after SGLT2I/DPP4I use were excluded.

**Table 5:** Subgroup analysis: Treatment effects of SGLT2I vs. DPP4I for incident adverse cognitive dysfunction events, and mortality outcomes in patients with both type−2 diabetes mellitus and cardiovascular diseases before and after propensity score matching (1:2).

**Outcome**	**Before matching (*N =* 39,828)**	**After matching (*N =* 49,830)**
	**[95% CI]; *P*-value**	**[95% CI]; *P*-value**
All–cause mortality	0.60 [0.55–0.65]; <0.0001^***^	0.45 [0.41–0.51]; <0.0001^***^
Cardiovascular mortality	0.63 [0.51–0.77]; <0.0001^***^	0.47 [0.33–0.65]; <0.0001^***^
Cerebrovascular mortality	0.40 [0.24–0.65]; 0.0003^***^	0.18 [0.15–0.22]; <0.0001^***^
New onset dementia	0.53 [0.42–0.68]; <0.0001^***^	0.20 [0.09–0.45]; 0.0001^***^
New onset Alzheimer's	0.62 [0.34–1.14]; 0.1262	0.27 [0.03–2.16]; 0.2155
New onset Parkinson's	0.77 [0.39–1.50]; 0.4429	0.42 [0.09–1.96]; 0.2706

After propensity-score matching, SGLT2I users had a median follow-up time of 459 days (IQR: 42–849) while DPP4I users had a median follow-up time of 522 days (IUQ: 74–1,004). SGLT2I users had a significantly lower risk of new-onset dementia (HR:0.2, 95% CI: [0.09, 0.45], *P* < 0.0001) but not new-onset Alzheimer's disease (HR:0.27, 95% CI: [0.03, 2.16], *P* = 0.2155) and Parkinson's disease (HR:0.42, 95% CI: [0.09, 1.96], *P* = 0.2706) compared with DPP4I users.

## Discussion

This study demonstrated several major findings. Firstly, SGLT2I users had a lower risk of new-onset dementia, Alzheimer's disease, and Parkinson's disease compared with DPP4I users. Secondly, SGLT2I users had a lower risk of all-cause mortality, as well as cerebrovascular and cardiovascular mortality. All of these were confirmed by univariate Cox regression analysis and competing risk analysis models apart from the association with Alzheimer's disease, which was not significantly reduced in SGLT2I users compared with DPP4I users.

The superior protective effect of DPP4I on dementia compared with other second-line anti-diabetic medication has been demonstrated by multiple studies (29–32). To our knowledge, no study so far has attempted a direct head-to-head between DPP4I and SGLT2I users for new-onset dementia, although a recent case-control study indirectly compared them when considering the risk of dementia associated with different antidiabetic medications (22). They found that while both DPP4I and SGLT2I were associated with lower odds of dementia, the odds ratio for dementia were 0.8 and 0.58 for DPP4I and SGLT2I, respectively. This is consistent with our findings that SGLT2I is superior to DPP4I in lowering dementia risk in diabetic patients. There are several possible explanations for the superior dementia-protective effects of SGLT2I. Firstly, both obesity and diabetes are independent risk factors for dementia due to shared pathophysiological mechanisms such as oxidative stress, inflammation, and insulin resistance (33, 34). Therefore, the increased reduction in weight and HbA1c observed in SGLT2I compared with DPP4I may account for the greater reduction in dementia risk (35, 36). Secondly, animal studies have proposed different neuroprotective mechanisms of SGLT2I and DPP4I which may account for their different efficacy in reducing dementia risk. DPP4I predominantly reduced amyloid deposition, tau phosphorylation, while increased GLP-1 and stromal-derived factor-1 which promoted neurogenesis (16, 37). In contrast, SGLT2I improved brain mitochondrial function, hippocampal synaptic plasticity and inhibited acetylcholinesterase (18, 20, 38).

Alzheimer's disease and diabetes are closely linked by mechanisms such as oxidative stress, amyloid deposition, and tau hyperphosphorylation, so much so that some have termed Alzheimer's as “Type-3 diabetes” (39, 40). There has been growing interest in DPP4I as a potential new therapy against Alzheimer's, with animal studies showing that it reduces amyloid β protein, tau phosphorylation, inflammatory cytokines, and neuronal cell apoptosis in the brain (37, 41–43). This is consistent with clinical studies which found that DPP4I use is associated with the reduced rate of memory decline and increased mini-mental state examination (MMSE) score in Alzheimer's patients compared with metformin use (44, 45). Research on the role of SGLT2I in Alzheimer's disease so far has been based predominantly on animal models, with promising studies suggesting that SGLT2 reduces the amyloid burden, tau pathology, and brain atrophy volume (46). Our finding that SGLT2I use was associated with lower or similar risks of Alzheimer's compared with DPP4I suggested that both may have potential roles as novel therapeutic approaches for Alzheimer's patients and the role of SGLT2I in Alzheimer's should be further explored. The subgroup analysis on patients with both type 2 diabetes and cardiovascular disease showed SGLT2I did not significantly reduce the risk of Alzheimer's disease and Parkinson's disease. This could be a reflection of the equally strong association between cardiovascular disease and such cognitive pathologies (47, 48), as well as their link with type-2 diabetes.

Parkinson's disease is another neurodegenerative disorder closely associated with diabetes, sharing pathophysiological mechanisms such as insulin dysregulation, amyloid deposition, microglial activation, and mitochondrial dysfunction (49). This has been confirmed clinically by several cohort studies which demonstrate type 2 diabetes is associated with an increased risk of Parkinson's (50, 51). Whilst the interest in this is much lower than that of Alzheimer's, several recent studies have suggested beneficial effects of DPP4I in diabetic patients with Parkinson's. A retrospective longitudinal cohort study found a strong protective association between DPP4I and GLP-1 agonist use and Parkinson's disease while another retrospective study found that DPP4I use was associated with increased dopamine transporter availability, slower increase in levodopa dose, and lower risk of levodopa-induced dyskinesia in diabetic patients with Parkinson's disease (52, 53). Our study is the first to compare DPP4I and SGLT2I in their associated Parkinson's risk and demonstrated that SGLT2I has superior protective effects against Parkinson's. Due to the close association and overlapping pathophysiology between Parkinson's disease and dementia with Lewy bodies, it could be inferred that SGLT2I also has superior protective effects against dementia with Lewy Bodies and Parkinson's disease dementia compared with DPP4I (54–56). To date, no study has examined the role of SGLT2I in Parkinson's disease or dementia with Lewy Bodies and our finding suggests that this is an exciting area of research that warrants further investigation.

### Limitations

Several limitations should be noted for the present study. First, given its observational nature, there was inherent information bias due to under-coding, coding errors, and missing data. Additionally, the drug compliance of the patient can only be assessed indirectly through prescription refills, which were ultimately not a direct measurement of drug exposure. Second, residual and post-baseline confounding might be present despite robust propensity-matching, particularly with the unavailability of information on lifestyle cardiovascular risk factors, e.g., smoking. The drug exposure duration among the patients has not been controlled, which might affect their risk against the study outcomes. Finally, the occurrence of cognitive dysfunction outcomes out of the hospital was not accounted for.

## Conclusions

The use of SGLT2I is associated with a significantly lower risk of dementia, Parkinson's disease, all-cause mortality, cardiovascular mortality, and cerebrovascular mortality compared with DPP4 use.

## Data Availability Statement

The original contributions presented in the study are included in the article/Supplementary Material, further inquiries can be directed to the corresponding author/s.

## Ethics Statement

The studies involving human participants were reviewed and approved by the Joint Chinese University of Hong Kong–New Territories East Cluster Clinical Research Ethics Committee. Written informed consent for participation was not required for this study in accordance with the national legislation and the institutional requirements.

## Author Contributions

JM and JZ: conception of study and literature search, preparation of figures, study design, data collection, data contribution, statistical analysis, data interpretation, manuscript drafting, and critical revision of manuscript. SL, KL, TLe, OC, ST, AW, TLi, WW, CC, GT, and QZ: conception of study and literature search, study design, data collection, data analysis, data contribution, manuscript drafting, critical revision of manuscript, and study supervision. All authors contributed to the article and approved the submitted version.

## Funding

This study was supported by the National Natural Science Foundation of China (NSFC) Grant Nos. 72042018, 71972164, and 71672163, in part by the Health and Medical Research Fund Grant (HMRF), the Food and Health Bureau, the Government of the Hong Kong Special Administrative Region No. 16171991, and in part by the Theme-Based Research Scheme of the Research Grants Council of Hong Kong Grant No. T32-102/14N.

## Conflict of Interest

The authors declare that the research was conducted in the absence of any commercial or financial relationships that could be construed as a potential conflict of interest.

## Publisher's Note

All claims expressed in this article are solely those of the authors and do not necessarily represent those of their affiliated organizations, or those of the publisher, the editors and the reviewers. Any product that may be evaluated in this article, or claim that may be made by its manufacturer, is not guaranteed or endorsed by the publisher.
