# Supplementary material for: Sodium-Glucose Cotransporter 2 (SGLT2) Inhibitors vs. Dipeptidyl Peptidase-4 (DPP4) Inhibitors for New-Onset Dementia: A Propensity Score-Matched Population-Based Study With Competing Risk Analysis
Source: Front Cardiovasc Med. 2021 Oct 21;8:747620. doi: 10.3389/fcvm.2021.747620 (PMC8566991; doi:10.3389/fcvm.2021.747620)
Supplement: Supplementary file 1 [file Data_Sheet_1.PDF]

Supplementary Table 1. ICD9 codes for comorbidities and ICD10 codes for outcomes.

Diabetes mellitus	250 250.01 250.02 250.03 250.1 250.11 250.12 250.13 250.2 250.21 250.22 250.23 250.3 250.31 250.32 250.33 250.4 250.41 250.42 250.43 250.5 250.51 250.52 250.53 250.6 250.61 250.62 250.63 250.7 250.71 250.72 250.73 250.8 250.81 250.82 250.83 250.9 250.91 250.92 250.93
Systemic embolism	444 444.01 444.09 444.1 444.2 444.21 444.22 444.8 444.81 444.89 444.9 445 445.01 445.02 445.8 445.81 445.89
Renal diseases	582 582 582.1 582.2 582.4 582.8 582.81 582.89 582.9 583 583 583.1 583.2 583.4 583.6 583.7 585 585.1 585.2 585.3 585.4 585.5 585.6 585.9 586 588 588 588.1 588.8 588.81 588.89 588.9
Acute myocardial infarction	410 410.01 410.02 410.1 410.11 410.12 410.2 410.21 410.22 410.3 410.31 410.32 410.4 410.41 410.42 410.5 410.51 410.52 410.6 410.61 410.62 410.7 410.71 410.72 410.8 410.81 410.82 410.9 410.91 410.92
Hypertension	401 401.1 401.9 402 402.01 402.1 402.11 402.9 402.91 403 403.01 403.1 403.11 403.9 403.91 404 404.01 404.02 404.03 404.1 404.11 404.12 404.13 404.9 404.91 404.92 404.93 405 405.01 405.09 405.1 405.11 405.19 405.9 405.91 405.99 437.2
Heart failure	428 428 428.1 428.2 428.2 428.21 428.22 428.23 428.3 428.3 428.31 428.32 428.33 428.4 428.4 428.41 428.42 428.43 428.9 398.91 402.01 402.11 402.91 404.01 404.03 404.11 404.13 404.91 404.93
Atrial fibrillation	427.31 429.4
Liver diseases	456 456.1 456.2 572.2 572.3 572.4 572.8 571.4 571.5 571.6
Dementia	331.82 290 290.1 290.11 290.12 290.13 290.2 290.21 290.3 290.4 290.41 290.42 290.43 290.8 290.9 294.2 294.1 294.11 294.21 332 46.1 333.4 340 42331 331.19 294.29
Chronic obstructive pulmonary disease	490 491 492 493 494 495 496 491.1 491.2 491.21 491.22 491.8 491.9 492.8 493.01 493.02 493.1 493.11 493.12 493.2 493.21 493.22 493.8 493.81 493.82 493.9 493.91 493.92 494.1 495.1 495.2 495.3 495.4 495.5 495.6 495.7 495.8 495.9
Peripheral vascular disease	250.7 443.9 443 443.1 443.2 443.21 443.22 443.23 443.24 443.29 443.8 443.81 443.82 443.89 441 443.9 785.4 V43.4
Stroke/transient ischemic attack	435 435.1 435.2 435.3 435.8 435.9 433.81 433.91 434 436 437 437.1 433.31 433.01 434.01 434.1

	434.11 434.9 434.91 437.2 437.3 437.4 437.5 437.6 437.7 437.8 437.9 430 431 432 432.1 432.9
Gastrointestinal bleeding	531 531.2 531.4 531.6 532 532.2 532.4 532.6 533 533.2 533.4 533.6 534 534.2 534.4 534.6 535.01 535.11 535.21 535.31 535.41 535.51 535.61 535.71 562.02 562.03 562.12 562.13 569.3 569.85 569.86 578 578.1 578.9
History of falls	781.2 781.3 781.99 V15.88 E880 E880.0 E880.1 E880.9 E881 E881.0 E881.1 E882 E883 E883.0 E883.1 E883.2 E883.9 E884 E884.0 E884.1 E884.2 E884.3 E884.4 E884.5 E884.6 E884.9 E885 E885.0 E885.1 E885.2 E885.3 E885.4 E885.9 E886 E886.0 E886.9 E887 E888 E888.0 E888.1 E888.8 E888.9
Pneumonia And Influenza	480 480.1 480.2 480.3 480.8 480.9 481 482 482.1 482.2 482.3 482.31 482.32 482.39 482.4 482.41 482.42 482.49 482.8 482.81 482.82 482.83 482.84 482.89 482.9 483 483.1 483.8 484 484.1 484.3 484.5 484.6 484.7 484.8 485 486 487 487.1 487.8 488 488.01 488.02 488.09 488.1 488.11 488.12 488.19 488.8 488.81 488.82 488.89
Ischemic heart disease	410.01 410.02 410.1 410.11 410.12 410.2 410.21 410.22 410.3 410.31 410.32 410.4 410.41 410.42 410.5 410.51 410.52 410.6 410.61 410.62 410.7 410.71 410.72 410.8 410.81 410.82 410.9 410.91 410.92 411 411.1 411.8 411.81 411.89 413 413.1 413.9 414 414.01 414.02 414.03 414.04 414.05 414.06 414.07 414.1 414.11 414.12 414.19 414.2 414.3 414.4 414.8 414.9 410 412
Malignancy	140 140.1 140.3 140.4 140.5 140.6 140.8 140.9 141 141.1 141.2 141.3 141.4 141.5 141.6 141.8 141.9 142 142.1 142.2 142.8 142.9 143 143.1 143.8 143.9 144 144.1 144.8 144.9 145 145.1 145.2 145.3 145.4 145.5 145.6 145.8 145.9 146 146.1 146.2 146.3 146.4 146.5 146.6 146.7 146.8 146.9 147 147.1 147.2 147.3 147.8 147.9 148 148.1 148.2 148.3 148.8 148.9 149 149.1 149.8 149.9 150 150.1 150.2 150.3 150.4 150.5 150.8 150.9 151 151.1 151.2 151.3 151.4 151.5 151.6 151.8 151.9 152 152.1 152.2 152.3 152.8 152.9 153 153.1 153.2 153.3 153.4 153.5 153.6 153.7 153.8 153.9 154 154.1 154.2 154.3 154.8 155 155.1 155.2 156 156.1 156.2 156.8 156.9 157 157.1 157.2 157.3 157.4 157.8 157.9 158 158.8 158.9 159 159.1 159.8 159.9 160 160.1 160.2 160.3 160.4 160.5 160.8 160.9 161 161.1 161.2 161.3 161.8 161.9 162 162.2 162.3 162.4 162.5 162.8 162.9 163 163.1 163.8 163.9 164 164.1 164.2 164.3 164.8 164.9 165 165.8 165.9 170 170.1 170.2 170.3 170.4 170.5 170.6 170.7 170.8 170.9 171 171.2 171.3 171.4 171.5 171.6 171.7 171.8 171.9 172 172.1 172.2 172.3 172.4 172.5 172.6 172.7 172.8 172.9

173 173.01 173.02 173.09 173.1 173.11 173.12 173.19 173.2
173.21 173.22 173.29 173.3 173.31 173.32 173.39 173.4 173.41
173.42 173.49 173.5 173.51 173.52 173.59 173.6 173.61 173.62
173.69 173.7 173.71 173.72 173.79 173.8 173.81 173.82 173.89
173.9 173.91 173.92 173.99 174 174.1 174.2 174.3 174.4
174.5 174.6 174.8 174.9 175 175.9 176 176.1 176.2 176.3
176.4 176.5 176.8 176.9 179 180 180.1 180.8 180.9 181 182
182.1 182.8 183 183.2 183.3 183.4 183.5 183.8 183.9 184
184.1 184.2 184.3 184.4 184.8 184.9 185 186 186.9 187
187.1 187.2 187.3 187.4 187.5 187.6 187.7 187.8 187.9
188 188.1 188.2 188.3 188.4 188.5 188.6 188.7 188.8
188.9 189 189.1 189.2 189.3 189.4 189.8 189.9 190 190.1
190.2 190.3 190.4 190.5 190.6 190.7 190.8 190.9 191
191.1 191.2 191.3 191.4 191.5 191.6 191.7 191.8 191.9
192 192.1 192.2 192.3 192.8 192.9 193 194 194.1 194.3
194.4 194.5 194.6 194.8 194.9 195 195.1 195.2 195.3
195.4 195.5 195.8 200 200.01 200.02 200.03 200.04 200.05
200.06 200.07 200.08 200.1 200.11 200.12 200.13 200.14 200.15
200.16 200.17 200.18 200.2 200.21 200.22 200.23 200.24 200.25
200.26 200.27 200.28 200.3 200.31 200.32 200.33 200.34 200.35
200.36 200.37 200.38 200.4 200.41 200.42 200.43 200.44 200.45
200.46 200.47 200.48 200.5 200.51 200.52 200.53 200.54 200.55
200.56 200.57 200.58 200.6 200.61 200.62 200.63 200.64 200.65
200.66 200.67 200.68 200.7 200.71 200.72 200.73 200.74 200.75
200.76 200.77 200.78 200.8 200.81 200.82 200.83 200.84 200.85
200.86 200.87 200.88 201 201.01 201.02 201.03 201.04 201.05
201.06 201.07 201.08 201.1 201.11 201.12 201.13 201.14 201.15
201.16 201.17 201.18 201.2 201.21 201.22 201.23 201.24 201.25
201.26 201.27 201.28 201.4 201.41 201.42 201.43 201.44 201.45
201.46 201.47 201.48 201.5 201.51 201.52 201.53 201.54 201.55
201.56 201.57 201.58 201.6 201.61 201.62 201.63 201.64 201.65
201.66 201.67 201.68 201.7 201.71 201.72 201.73 201.74 201.75
201.76 201.77 201.78 201.9 201.91 201.92 201.93 201.94 201.95
201.96 201.97 201.98 202 202.01 202.02 202.03 202.04 202.05
202.06 202.07 202.08 202.1 202.11 202.12 202.13 202.14 202.15
202.16 202.17 202.18 202.2 202.21 202.22 202.23 202.24 202.25
202.26 202.27 202.28 202.3 202.31 202.32 202.33 202.34 202.35
202.36 202.37 202.38 202.4 202.41 202.42 202.43 202.44 202.45
202.46 202.47 202.48 202.5 202.51 202.52 202.53 202.54 202.55
202.56 202.57 202.58 202.6 202.61 202.62 202.63 202.64 202.65
202.66 202.67 202.68 202.7 202.71 202.72 202.73 202.74 202.75
202.76 202.77 202.78 202.8 202.81 202.82 202.83 202.84 202.85
202.86 202.87 202.88 202.9 202.91 202.92 202.93 202.94 202.95
202.96 202.97 202.98 203 203.01 203.02 203.1 203.11 203.12

	203.8 203.81 203.82 204 204.01 204.02 204.1 204.11 204.12 204.2 204.21 204.22 204.8 204.81 204.82 204.9 204.91 204.92 205 205.01 205.02 205.1 205.11 205.12 205.2 205.21 205.22 205.3 205.31 205.32 205.8 205.81 205.82 205.9 205.91 205.92 206 206.01 206.02 206.1 206.11 206.12 206.2 206.21 206.22 206.8 206.81 206.82 206.9 206.91 206.92 207 207.01 207.02 207.1 207.11 207.12 207.2 207.21 207.22 207.8 207.81 207.82 208 208.01 208.02 208.1 208.11 208.12 208.2 208.21 208.22 208.8 208.81 208.82 208.9 208.91 208.92
Metastatic solid tumor	196 196 196.1 196.2 196.3 196.5 196.6 196.8 196.9 197 197 197.1 197.2 197.3 197.4 197.5 197.6 197.7 197.8 198 198 198.1 198.2 198.3 198.4 198.5 198.6 198.7 198.8 198.81 198.82 198.89 199 199 199.1

Supplementary Table 2. Confounding balancing comparisons of treated (SGLT2I) and controls (DPP4I) after propensity score matching with 1:2 nearest neighbor search.

	Coef.	Std.Err.	z	P>z	[95%Conf.	Interval]
Male gender	0.074	0.019	3.850	0.000	0.036	0.112
Baseline age, years	-0.018	0.001	-14.790	0.000	-0.020	-0.015
Charlson comorbidity index	-0.127	0.009	-14.360	0.000	-0.145	-0.110
Non-SGLT2I/DPP4I drugs	1.638	0.273	6.010	0.000	1.104	2.172
Other anti-diabetic drugs	-0.463	0.046	-9.960	0.000	-0.554	-0.372
Fast glucose, mmol/L	0.005	0.002	2.450	0.014	0.001	0.010
HbA1c, g/dL	0.018	0.002	9.570	0.000	0.014	0.022
Constant	-0.566	0.278	-2.040	0.042	-1.110	-0.021

Variable	Sample	Treated	Controls	Difference	S.E.	T-stat
mortality	Unmatched	0.067	0.189	-0.121	0.005	-22.390
ATT		0.067	0.109	-0.042	0.006	-7.120

Note: S.E. does not take into account that the propensity score is estimated.

Supplementary Table 3. Univariate Cox analyses of significant risk factors for new-onset dementia, Alzheimer's and Parkinson's before and after propensity score matching (1:2).

* for p≤ 0.05, ** for p ≤ 0.01, *** for p ≤ 0.001; SGLT2I: Sodium-glucose cotransporter-2 inhibitors; DPP4I: Dipeptidyl peptidase-4 inhibitors; NLR: neutrophil-to-lymphocyte ratio; TIA: transient ischemic attack.

Characteristics	Before matching			After matching		
	**New onset dementia HR [95% CI];P value**	**New onset Alzheimer HR [95% CI];P value**	**New onset Parkinson HR [95% CI];P value**	**New onset dementia HR [95% CI];P value**	**New onset Alzheimer HR [95% CI];P value**	**New onset Parkinson HR [95% CI];P value**
Demographics						
Male gender	0.59[0.51-0.68];<0.0001***	0.43[0.29-0.64];<0.0001***	1.20[0.76-1.88];0.4365	0.82[0.63-1.06];0.1339	0.46[0.22-0.96];0.0397*	1.63[0.84-3.19];0.1507
Female gender	1.70[1.47-1.97];<0.0001***	2.33[1.56-3.48];<0.0001***	0.84[0.53-1.31];0.4365	1.22[0.94-1.58];0.1339	2.17[1.04-4.55];0.0397*	0.61[0.31-1.20];0.1507
Baseline age, year	1.12[1.11-1.13];<0.0001***	1.14[1.12-1.17];<0.0001***	1.08[1.06-1.10];<0.0001***	1.13[1.12-1.15];<0.0001***	1.19[1.15-1.24];<0.0001***	1.09[1.06-1.12];<0.0001***
<40	1.0[Reference]	1.0[Reference]	1.0[Reference]	1.0[Reference]	1.0[Reference]	1.0[Reference]
[40, 50)	0.02[0.00-0.13];0.0001***	-	-	0.09[0.03-0.27];<0.0001***	-	-
[50-60)	0.06[0.04-0.11];<0.0001***	-	0.24[0.10-0.60];0.0022**	0.11[0.06-0.21];<0.0001***	-	0.45[0.20-1.01];0.0516
[60-70)	0.27[0.22-0.35];<0.0001***	0.09[0.03-0.23];<0.0001***	0.54[0.30-0.94];0.0302*	0.63[0.47-0.86];0.0030**	0.24[0.07-0.80];0.0198*	0.95[0.50-1.83];0.8841
[70-80)	1.37[1.16-1.61];0.0002***	1.31[0.86-2.01];0.2140	1.91[1.20-3.05];0.0068**	2.46[1.85-3.28];<0.0001***	2.50[1.11-5.65];0.0276*	3.78[2.01-7.12];<0.0001***
>=80	8.15[7.02-9.45];<0.0001***	12.47[8.26-18.81];<0.0001***	3.61[2.28-5.71];<0.0001***	15.15[11.68-19.65];<0.0001***	34.05[16.03-72.36];<0.0001***	4.30[1.90-9.70];0.0004***
Charlson score	1.44[1.40-1.48];<0.0001***	1.46[1.36-1.56];<0.0001***	1.33[1.21-1.47];<0.0001***	1.63[1.56-1.71];<0.0001***	1.71[1.53-1.92];<0.0001***	1.48[1.30-1.69];<0.0001***

NLR	1.02[1.01-1.03];<0.0001***	1.03[1.01-1.05];0.0090**	1.03[1.02-1.05];0.0002***	1.03[1.01-1.04];0.0005***	1.03[1.00-1.06];0.0510	1.04[1.03-1.06];<0.0001***
Past comorbidities						
Hypertension	2.44[2.09-2.84];<0.0001***	1.42[0.91-2.21];0.1218	1.84[1.12-3.03];0.0160*	2.82[2.14-3.70];<0.0001***	1.93[0.82-4.52];0.1311	2.86[1.48-5.51];0.0017**
Heart failure	3.04[2.06-4.50];<0.0001***	2.40[0.76-7.56];0.1356	1.14[0.16-8.23];0.8937	2.28[0.94-5.54];0.0679	3.87[0.53-28.47];0.1837	-
Renal diseases	1.81[1.40-2.34];<0.0001***	1.30[0.60-2.80];0.5045	1.05[0.38-2.88];0.9233	3.19[1.89-5.39];<0.0001***	3.61[0.86-15.18];0.0803	2.51[0.61-10.42];0.2036
Liver diseases	0.71[0.23-2.22];0.5613	-	2.43[0.34-17.47];0.3782	-	-	7.31[1.00-53.24];0.0495*
Stroke/TIA	2.27[1.64-3.14];<0.0001***	1.97[0.80-4.83];0.1401	0.55[0.08-3.93];0.5473	2.18[1.19-3.99];0.0118*	-	-
Gastrointestinal bleeding	2.48[1.70-3.63];<0.0001***	2.40[0.88-6.52];0.0863	2.62[0.83-8.32];0.1021	3.50[1.80-6.82];0.0002***	-	4.73[1.14-19.59];0.0322*
History of falls	3.85[3.19-4.64];<0.0001***	3.85[2.37-6.27];<0.0001***	3.23[1.74-5.99];0.0002***	4.85[3.42-6.86];<0.0001***	4.17[1.45-12.00];0.0082**	3.71[1.46-9.48];0.0060**
Pneumonia and influenza	3.88[2.92-5.16];<0.0001***	3.58[1.66-7.71];0.0011**	2.97[1.08-8.14];0.0345*	6.29[3.78-10.46];<0.0001***	18.99[7.20-50.07];<0.0001***	4.94[1.19-20.54];0.0279*
Endocrine	1.52[0.99-2.32];0.0530	1.88[0.69-5.10];0.2167	0.64[0.09-4.59];0.6550	1.60[0.71-3.60];0.2553	4.46[1.06-18.78];0.0412*	4.64[1.43-15.01];0.0105*
Atrial fibrillation	2.00[1.49-2.69];<0.0001***	1.10[0.41-3.00];0.8461	-	2.55[1.42-4.56];0.0016**	1.77[0.24-13.00];0.5766	-
Ischemic heart disease	1.60[1.29-2.00];<0.0001***	1.55[0.87-2.77];0.1405	0.80[0.32-1.98];0.6245	2.05[1.41-3.00];0.0002***	2.23[0.78-6.43];0.1364	0.35[0.05-2.57];0.3048
Peripheral vascular disease	1.92[1.08-3.39];0.0254*	1.09[0.15-7.83];0.9296	3.24[0.79-13.20];0.1013	0.63[0.09-4.49];0.6452	-	-
Malignancy	1.00[0.60-1.66];0.9903	0.46[0.06-3.26];0.4334	0.67[0.09-4.79];0.6859	3.48[1.72-7.05];0.0005***	-	-
Metastatic solid tumor	0.98[0.32-3.06];0.9775	-	3.57[0.49-25.71];0.2072	5.01[1.25-20.18];0.0233*	-	-
Medications						

SGLT2I v.s. DPP4I	0.53[0.42-0.68];<0.0001***	0.62[0.34-1.14];0.1262	0.77[0.39-1.50];0.4429	0.41[0.27-0.61];<0.0001***	0.25[0.06-1.04];0.0569	0.28[0.09-0.91];0.0349*
Beta blockers	0.07[0.01-0.48];0.0072**	-	-	0.09[0.01-0.65];0.0171*	-	-
Diuretics	0.00[0.00-Inf];0.9839	-	-	-	-	-
Anticoagulants	0.60[0.28-1.26];0.1780	0.63[0.09-4.51];0.6458	0.45[0.06-3.21];0.4222	-	-	-
Antiplatelets	0.06[0.02-0.25];0.0001***	-	-	0.06[0.01-0.42];0.0048**	-	-
Antihypertensive drugs	0.21[0.05-0.85];0.0285*	-	-	0.48[0.12-1.94];0.3039	-	-
Lipid-lowering drugs	0.06[0.03-0.15];<0.0001***	0.09[0.01-0.62];0.0148*	-	0.09[0.04-0.26];<0.0001***	0.20[0.03-1.51];0.1192	-
Statins and fibrates	0.77[0.61-0.97];0.0235*	0.60[0.31-1.16];0.1276	0.96[0.51-1.82];0.8972	0.62[0.42-0.94];0.0236*	0.58[0.17-1.90];0.3660	1.57[0.77-3.19];0.2145
Non-steroidal anti-inflammatory drugs	0.07[0.02-0.27];0.0001***	-	-	0.06[0.01-0.45];0.0057**	-	-
Other antidiabetic drugs	1.41[1.02-1.94];0.0379*	1.13[0.53-2.44];0.7510	1.10[0.45-2.73];0.8328	1.48[0.97-2.26];0.0678	4.68[0.64-34.38];0.1297	1.19[0.47-3.03];0.7130
Complete blood counts						
Mean corpuscular volume, fL	1.01[1.00-1.03];0.0447*	1.01[0.97-1.05];0.5568	0.98[0.95-1.01];0.2431	1.07[1.04-1.10];<0.0001***	1.10[1.01-1.19];0.0222*	1.00[0.95-1.05];0.8873
Basophil, x10^9/L	0.01[0.00-0.22];0.0041**	0.72[0.00-1255.74];0.9310	1.09[0.01-123.27];0.9705	0.00[0.00-0.86];0.0445*	1.33[0.06-27.68];0.8547	0.16[0.00-84406.93];0.7836
Eosinophil, x10^9/L	0.90[0.57-1.40];0.6331	0.93[0.27-3.19];0.9041	0.69[0.12-3.87];0.6765	1.35[0.64-2.89];0.4325	0.69[0.03-14.49];0.8101	0.00[0.00-0.09];0.0041**
Lymphocyte, x10^9/L	0.52[0.45-0.60];<0.0001***	0.44[0.28-0.68];0.0003***	0.37[0.22-0.63];0.0002***	0.38[0.29-0.50];<0.0001***	0.26[0.11-0.62];0.0024**	0.18[0.09-0.37];<0.0001***
Monocyte, x10^9/L	0.79[0.50-1.25];0.3098	1.25[0.41-3.79];0.6953	1.68[0.58-4.83];0.3367	0.80[0.34-1.87];0.6051	2.76[0.55-13.99];0.2197	2.98[0.84-10.58];0.0918

Neutrophil, x10^9/L	1.04[1.01-1.07];0.0102*	1.06[1.00-1.13];0.0496*	1.07[1.01-1.14];0.0152*	1.03[0.98-1.09];0.2690	1.07[0.98-1.17];0.1153	1.10[1.05-1.14];<0.0001***
White blood count, x10^9/L	1.01[0.99-1.03];0.4309	1.02[0.98-1.06];0.3632	0.98[0.88-1.10];0.7823	1.00[0.95-1.06];0.9617	1.02[0.95-1.09];0.5965	1.02[0.98-1.07];0.3498
Mean cell haemoglobin, pg	1.02[0.98-1.05];0.3474	1.02[0.93-1.12];0.6654	0.99[0.90-1.09];0.7941	1.14[1.06-1.23];0.0004***	1.17[0.94-1.46];0.1622	1.05[0.91-1.21];0.5136
Platelet, x10^9/L	0.999[0.998-1.000];0.1003	1.00[0.99-1.00];0.0977	1.000[0.996-1.004];0.8948	1.00[0.99-1.00];0.0048**	0.99[0.98-0.99];0.0014**	1.00[1.00-1.01];0.6531
Red blood count, x10^12/L	0.47[0.41-0.54];<0.0001***	0.63[0.43-0.92];0.0174*	0.61[0.40-0.93];0.0217*	0.28[0.22-0.35];<0.0001***	0.29[0.15-0.57];0.0003***	0.51[0.29-0.91];0.0225*
Liver and renal biochemical tests						
K/Potassium, mmol/L	1.07[0.91-1.25];0.4303	0.69[0.44-1.08];0.1018	1.53[0.95-2.46];0.0826	1.10[0.80-1.49];0.5627	0.77[0.32-1.88];0.5726	0.95[0.47-1.93];0.8949
Urate, mmol/L	2.34[0.39-13.98];0.3498	0.18[0.00-63.85];0.5651	0.01[0.00-1.38];0.0664	0.41[0.02-9.54];0.5758	0.01[0.00-195.27];0.3668	0.01[0.00-5.53];0.1593
Albumin, g/L	0.90[0.89-0.92];<0.0001***	0.92[0.88-0.97];0.0013**	0.91[0.86-0.97];0.0013**	0.88[0.86-0.91];<0.0001***	0.88[0.81-0.96];0.0025**	0.95[0.87-1.02];0.1691
Na/Sodium, mmol/L	0.97[0.94-0.99];0.0071**	1.05[0.98-1.14];0.1769	0.91[0.85-0.97];0.0037**	0.98[0.93-1.03];0.3563	1.05[0.91-1.22];0.4842	0.91[0.83-1.00];0.0620
Urea, mmol/L	1.05[1.03-1.06];<0.0001***	1.02[0.97-1.07];0.4080	1.03[0.98-1.08];0.2131	1.07[1.05-1.10];<0.0001***	1.06[0.98-1.15];0.1642	1.04[0.96-1.12];0.3605
Protein, g/L	0.96[0.94-0.97];<0.0001***	0.96[0.92-1.00];0.0311*	0.95[0.90-1.00];0.0320*	0.94[0.91-0.96];<0.0001***	0.96[0.88-1.04];0.3099	0.97[0.91-1.04];0.3805
Creatinine, umol/L	1.001[1.001-1.002];<0.0001***	1.000[0.998-1.002];0.7056	1.000[0.998-1.003];0.8547	1.002[1.001-1.003];0.0001***	1.002[0.999-1.004];0.2152	1.001[0.998-1.004];0.4522
Alkaline phosphatase, U/L	1.003[1.002-1.004];<0.0001***	1.00[0.99-1.01];0.7232	1.00[1.00-1.01];0.1380	1.00[1.00-1.01];0.0017**	1.00[0.99-1.02];0.9498	1.00[1.00-1.01];0.1291
Aspartate transaminase, U/L	0.99[0.98-1.00];0.1002	0.99[0.95-1.02];0.5176	1.00[0.97-1.02];0.8297	1.00[0.99-1.01];0.9216	0.89[0.75-1.05];0.1557	0.95[0.88-1.03];0.2387
Alanine transaminase, U/L	0.97[0.97-0.98];<0.0001***	0.95[0.92-0.98];0.0003***	1.00[0.99-1.01];0.6848	0.94[0.92-0.96];<0.0001***	0.89[0.83-0.95];0.0006***	1.01[1.00-1.01];0.0853

Bilirubin, umol/L	0.99[0.97-1.00];0.0941	0.94[0.89-0.99];0.0304*	1.00[0.95-1.04];0.8988	0.99[0.96-1.02];0.4391	0.93[0.84-1.04];0.1920	1.02[1.00-1.04];0.0995
Glycemic and lipid profiles						
Triglyceride, mmol/L	0.88[0.81-0.96];0.0038**	0.66[0.47-0.91];0.0112*	0.86[0.66-1.12];0.2668	0.66[0.54-0.81];0.0001***	0.67[0.37-1.21];0.1866	0.95[0.72-1.25];0.6920
Total cholesterol, mmol/L	0.91[0.85-0.97];0.0035**	0.79[0.66-0.94];0.0068**	0.82[0.68-0.99];0.0415*	0.91[0.81-1.02];0.0907	0.86[0.62-1.20];0.3798	0.79[0.62-1.02];0.0672
Low-density lipoprotein (LDL), mmol/L	0.89[0.79-1.01];0.0654	0.58[0.40-0.84];0.0039**	0.72[0.49-1.07];0.1044	0.98[0.80-1.21];0.8696	0.71[0.37-1.38];0.3139	0.61[0.35-1.08];0.0891
High-density lipoprotein (LDL), mmol/L	1.45[1.13-1.87];0.0039**	1.49[0.76-2.94];0.2448	1.98[1.01-3.87];0.0452*	2.81[1.86-4.26];<0.0001***	2.56[0.75-8.78];0.1351	2.93[1.12-7.67];0.0288*
Fast glucose, mmol/L	1.01[0.99-1.03];0.5042	0.94[0.86-1.02];0.1265	1.00[0.94-1.08];0.8932	1.03[0.99-1.06];0.1169	0.95[0.83-1.10];0.4906	1.00[0.91-1.09];0.9573
HbA1C, g/dL	0.97[0.96-0.99];0.0046**	1.01[0.95-1.06];0.8464	1.00[0.94-1.06];0.9489	0.99[0.96-1.03];0.6282	1.00[0.90-1.11];0.9693	1.02[0.94-1.10];0.6894

Supplementary Table 4. Univariate Cox analyses of significant risk factors for all-cause mortality, cardiovascular mortality and cerebrovascular mortality before and after propensity score matching (1:2).

* for p≤ 0.05, ** for p ≤ 0.01, *** for p ≤ 0.001; SGLT2I: Sodium-glucose cotransporter-2 inhibitors; DPP4I: Dipeptidyl peptidase-4 inhibitors; NLR: neutrophil-to-lymphocyte ratio; TIA: transient ischemic attack.

Characteristics	Before matching			After matching		
	All-cause mortality HR [95% CI];P value	Cardiovascular mortality HR [95% CI];P value	Cerebrovascular mortality HR [95% CI];P value	All-cause mortality HR [95% CI];P value	Cardiovascular mortality HR [95% CI];P value	Cerebrovascular mortality HR [95% CI];P value
Demographics						
Male gender	1.06[1.00-1.12];0.0347*	1.00[0.87-1.15];0.9920	1.03[0.79-1.36];0.8135	0.51[0.48-0.55];<0.0001***	1.20[0.97-1.49];0.0999	0.05[0.04-0.06];<0.0001***
Female gender	0.94[0.90-1.00];0.0347*	1.00[0.87-1.15];0.9920	0.97[0.74-1.27];0.8135	1.96[1.83-2.10];<0.0001***	0.83[0.67-1.04];0.0999	20.31[16.18-25.49];<0.0001***
Baseline age, year	1.081[1.079-1.084];<0.0001***	1.07[1.06-1.08];<0.0001***	1.07[1.05-1.08];<0.0001***	1.071[1.068-1.074];<0.0001***	1.07[1.06-1.08];<0.0001***	1.03[1.03-1.04];<0.0001***
<40	1.0[Reference]	1.0[Reference]	1.0[Reference]	1.0[Reference]	1.0[Reference]	1.0[Reference]
[40, 50)	0.20[0.16-0.24];<0.0001***	0.24[0.14-0.40];<0.0001***	0.51[0.25-1.04];0.0635	0.20[0.17-0.25];<0.0001***	0.40[0.26-0.63];0.0001***	0.08[0.05-0.13];<0.0001***
[50-60)	0.26[0.24-0.29];<0.0001***	0.36[0.28-0.46];<0.0001***	0.18[0.09-0.33];<0.0001***	0.24[0.21-0.26];<0.0001***	0.44[0.33-0.58];<0.0001***	0.03[0.02-0.05];<0.0001***
[60-70)	0.48[0.44-0.51];<0.0001***	0.48[0.40-0.57];<0.0001***	0.69[0.50-0.94];0.0207*	2.01[1.88-2.15];<0.0001***	0.89[0.71-1.12];0.3245	26.53[21.14-33.30];<0.0001***
[70-80)	1.30[1.22-1.38];<0.0001***	1.38[1.19-1.61];<0.0001***	1.18[0.86-1.62];0.3004	1.26[1.15-1.38];<0.0001***	2.02[1.59-2.58];<0.0001***	0.14[0.09-0.21];<0.0001***
>=80	4.90[4.65-5.17];<0.0001***	4.13[3.60-4.75];<0.0001***	4.01[3.04-5.28];<0.0001***	5.24[4.83-5.69];<0.0001***	6.21[4.87-7.92];<0.0001***	0.38[0.26-0.57];<0.0001***
Charlson score	1.54[1.53-1.56];<0.0001***	1.49[1.45-1.52];<0.0001***	1.43[1.36-1.51];<0.0001***	1.53[1.51-1.55];<0.0001***	1.60[1.54-1.66];<0.0001***	1.15[1.11-1.19];<0.0001***

NLR	1.02[1.02-1.03];<0.0001***	1.02[1.01-1.03];<0.0001***	1.03[1.01-1.04];<0.0001***	1.00[0.98-1.01];0.4159	1.03[1.01-1.04];<0.0001***	0.26[0.23-0.29];<0.0001***
Past comorbidities						
Hypertension	3.26[3.09-3.44];<0.0001***	3.47[3.02-3.99];<0.0001***	3.67[2.79-4.83];<0.0001***	2.05[1.90-2.21];<0.0001***	3.25[2.62-4.03];<0.0001***	0.28[0.21-0.36];<0.0001***
Heart failure	5.34[4.79-5.96];<0.0001***	7.31[5.70-9.37];<0.0001***	2.39[1.06-5.40];0.0352*	4.53[3.82-5.37];<0.0001***	7.90[5.26-11.87];<0.0001***	0.17[0.04-0.70];0.0138*
Renal diseases	4.93[4.62-5.26];<0.0001***	4.97[4.20-5.88];<0.0001***	4.40[3.12-6.22];<0.0001***	5.16[4.61-5.77];<0.0001***	8.18[6.11-10.96];<0.0001***	0.37[0.20-0.69];0.0019**
Liver diseases	4.63[3.92-5.45];<0.0001***	2.12[1.13-3.95];0.0184*	3.36[1.25-9.03];0.0165*	4.10[3.12-5.39];<0.0001***	1.50[0.37-6.03];0.5656	0.21[0.03-1.48];0.1171
Stroke/TIA	2.46[2.20-2.76];<0.0001***	2.22[1.64-3.01];<0.0001***	4.40[2.80-6.92];<0.0001***	1.73[1.45-2.07];<0.0001***	2.33[1.45-3.74];0.0005***	0.28[0.14-0.57];0.0004***
Gastrointestinal bleeding	2.95[2.60-3.34];<0.0001***	1.98[1.34-2.92];0.0006***	2.10[0.99-4.46];0.0541	2.50[2.05-3.06];<0.0001***	1.46[0.65-3.26];0.3611	0.20[0.07-0.63];0.0060**
History of falls	2.82[2.62-3.04];<0.0001***	2.49[2.04-3.05];<0.0001***	3.55[2.48-5.07];<0.0001***	2.38[2.11-2.68];<0.0001***	2.78[1.97-3.92];<0.0001***	0.20[0.11-0.37];<0.0001***
Pneumonia and influenza	4.24[3.84-4.68];<0.0001***	3.33[2.51-4.42];<0.0001***	1.23[0.51-2.98];0.6499	4.10[3.49-4.81];<0.0001***	6.45[4.29-9.70];<0.0001***	0.07[0.01-0.47];0.0067**
Endocrine	3.35[3.02-3.73];<0.0001***	1.20[0.77-1.86];0.4287	2.14[1.10-4.18];0.0253*	2.64[2.23-3.12];<0.0001***	0.83[0.34-2.01];0.6858	0.21[0.08-0.56];0.0017**
Atrial fibrillation	4.03[3.72-4.36];<0.0001***	6.28[5.26-7.50];<0.0001***	6.88[4.88-9.69];<0.0001***	3.91[3.44-4.44];<0.0001***	8.98[6.76-11.93];<0.0001***	0.72[0.46-1.13];0.1551
Ischemic heart disease	2.83[2.66-3.02];<0.0001***	6.33[5.49-7.30];<0.0001***	2.19[1.52-3.15];<0.0001***	1.91[1.73-2.11];<0.0001***	6.18[4.94-7.71];<0.0001***	0.21[0.13-0.33];<0.0001***
Peripheral vascular disease	4.58[3.99-5.24];<0.0001***	6.71[4.98-9.04];<0.0001***	3.33[1.48-7.51];0.0037**	3.84[3.11-4.75];<0.0001***	10.54[6.96-15.95];<0.0001***	0.58[0.24-1.39];0.2178
Malignancy	4.74[4.33-5.20];<0.0001***	1.65[1.13-2.41];0.0093**	2.12[1.09-4.14];0.0274*	4.33[3.67-5.11];<0.0001***	2.21[1.09-4.45];0.0270*	0.16[0.04-0.64];0.0097**
Metastatic solid tumor	12.68[11.25-14.30];<0.0001***	1.39[0.58-3.36];0.4604	4.54[1.69-12.24];0.0028**	13.10[10.43-16.45];<0.0001***	1.61[0.23-11.47];0.6339	-
Medications						

SGLT2I v.s. DPP4I	0.60[0.55-0.65];<0.0001***	0.63[0.51-0.77];<0.0001***	0.40[0.24-0.65];0.0003***	0.84[0.77-0.91];<0.0001***	0.64[0.49-0.85];0.0017**	0.36[0.30-0.43];<0.0001***
Beta blockers	0.44[0.34-0.58];<0.0001***	0.79[0.47-1.35];0.3925	0.44[0.11-1.78];0.2507	11.44[10.61-12.33];<0.0001***	0.85[0.50-1.46];0.5555	187.84[155.91-226.30];<0.0001***
Diuretics	0.46[0.36-0.60];<0.0001***	0.97[0.60-1.57];0.8992	0.66[0.21-2.08];0.4815	0.58[0.45-0.76];0.0001***	1.62[0.98-2.68];0.0599	0.07[0.02-0.27];0.0001***
Anticoagulants	0.59[0.46-0.77];0.0001***	1.19[0.45-3.19];0.7235	0.20[0.09-0.45];0.0001***	0.15[0.08-0.29];<0.0001***	-	-
Antiplatelets	0.39[0.32-0.48];<0.0001***	0.65[0.44-0.98];0.0375*	0.94[0.48-1.84];0.8608	6.90[6.42-7.42];<0.0001***	1.00[0.66-1.50];0.9946	126.06[103.02-154.26];<0.0001***
Antihypertensive drugs	0.46[0.33-0.64];<0.0001***	0.70[0.35-1.40];0.3138	0.34[0.05-2.43];0.2834	0.59[0.42-0.82];0.0015**	1.22[0.61-2.47];0.5720	0.05[0.01-0.35];0.0027**
Lipid-lowering drugs	0.21[0.18-0.25];<0.0001***	0.30[0.21-0.43];<0.0001***	0.48[0.27-0.87];0.0159*	2.99[2.78-3.21];<0.0001***	0.46[0.32-0.68];0.0001***	57.20[46.71-70.05];<0.0001***
Statins and fibrates	0.50[0.45-0.55];<0.0001***	0.47[0.36-0.60];<0.0001***	0.54[0.34-0.88];0.0135*	0.42[0.37-0.48];<0.0001***	0.61[0.44-0.84];0.0028**	0.05[0.03-0.09];<0.0001***
Non-steroidal anti-inflammatory drugs	0.40[0.33-0.49];<0.0001***	0.64[0.42-0.97];0.0369*	1.01[0.52-1.97];0.9797	7.29[6.77-7.83];<0.0001***	0.97[0.63-1.48];0.8823	133.06[108.74-162.83];<0.0001***
Other antidiabetic drugs	1.00[0.91-1.11];0.9444	1.03[0.79-1.34];0.8246	0.81[0.51-1.30];0.3847	1.73[1.53-1.94];<0.0001***	1.48[1.05-2.08];0.0236*	16.60[9.40-29.32];<0.0001***
Complete blood counts						
Mean corpuscular volume, fL	1.02[1.01-1.02];<0.0001***	1.01[1.00-1.03];0.0137*	1.01[0.98-1.03];0.6513	1.04[1.03-1.04];<0.0001***	1.00[0.99-1.02];0.6936	1.05[1.04-1.06];<0.0001***
Basophil, x10^9/L	0.15[0.06-0.42];0.0003***	0.62[0.06-6.58];0.6950	0.40[0.00-0.73];0.0383*	-	1.19[0.42-3.37];0.7476	0.000[0.000-0.000];<0.0001***
Eosinophil, x10^9/L	1.10[1.01-1.20];0.0292*	1.10[0.88-1.37];0.3921	1.14[0.81-1.61];0.4588	0.89[0.73-1.09];0.2734	1.33[0.79-2.26];0.2857	0.64[0.47-0.89];0.0075**
Lymphocyte, x10^9/L	0.56[0.53-0.59];<0.0001***	0.61[0.54-0.69];<0.0001***	0.57[0.44-0.73];<0.0001***	0.87[0.83-0.92];<0.0001***	0.57[0.47-0.68];<0.0001***	1.07[1.05-1.09];<0.0001***
Monocyte, x10^9/L	1.57[1.41-1.75];<0.0001***	1.55[1.18-2.05];0.0019**	1.61[0.94-2.75];0.0840	0.35[0.29-0.44];<0.0001***	2.20[1.44-3.35];0.0003***	0.04[0.03-0.06];<0.0001***

Neutrophil, x10^9/L	1.05[1.04-1.06];<0.0001***	1.05[1.02-1.07];<0.0001***	1.05[1.00-1.09];0.0440*	0.89[0.87-0.91];<0.0001***	1.06[1.03-1.09];<0.0001***	0.47[0.45-0.50];<0.0001***
White blood count, x10^9/L	1.02[1.01-1.02];<0.0001***	1.02[1.00-1.03];0.0092**	1.01[0.97-1.05];0.6196	0.93[0.91-0.94];<0.0001***	1.02[1.00-1.04];0.0531	0.79[0.76-0.81];<0.0001***
Mean cell haemoglobin, pg	1.02[1.01-1.03];0.0026**	1.01[0.98-1.04];0.4892	1.01[0.95-1.07];0.7581	1.10[1.08-1.12];<0.0001***	0.98[0.94-1.02];0.3825	1.22[1.19-1.25];<0.0001***
Platelet, x10^9/L	0.997[0.997-0.998];<0.0001***	0.998[0.997-0.999];0.0004***	1.00[0.99-1.00];0.0003***	0.999[0.998-0.999];<0.0001***	0.998[0.996-1.000];0.0307*	1.001[1.000-1.002];0.0311*
Red blood count, x10^12/L	0.46[0.44-0.48];<0.0001***	0.50[0.45-0.57];<0.0001***	0.51[0.41-0.64];<0.0001***	0.42[0.39-0.44];<0.0001***	0.58[0.48-0.70];<0.0001***	0.46[0.42-0.50];<0.0001***
Liver and renal biochemical tests						
K/Potassium, mmol/L	1.03[0.97-1.09];0.2854	1.09[0.94-1.27];0.2490	0.82[0.60-1.10];0.1870	2.07[1.93-2.23];<0.0001***	0.90[0.70-1.15];0.3968	4.32[3.99-4.67];<0.0001***
Urate, mmol/L	12.74[7.86-20.66];<0.0001***	72.33[25.81-202.70];<0.0001***	30.33[2.95-311.56];0.0041**	27.68[12.25-62.53];<0.0001***	329.86[55.31-1967.31];<0.0001***	605.36[8.95-40923.01];0.0029**
Albumin, g/L	0.87[0.87-0.88];<0.0001***	0.88[0.87-0.89];<0.0001***	0.91[0.88-0.94];<0.0001***	0.85[0.85-0.86];<0.0001***	0.86[0.84-0.88];<0.0001***	0.84[0.84-0.85];<0.0001***
Na/Sodium, mmol/L	0.94[0.93-0.95];<0.0001***	0.94[0.92-0.96];<0.0001***	0.91[0.87-0.95];<0.0001***	0.95[0.94-0.96];<0.0001***	0.93[0.90-0.96];<0.0001***	0.96[0.94-0.98];<0.0001***
Urea, mmol/L	1.08[1.08-1.09];<0.0001***	1.09[1.08-1.10];<0.0001***	1.08[1.07-1.10];<0.0001***	1.06[1.05-1.07];<0.0001***	1.11[1.09-1.12];<0.0001***	0.55[0.53-0.58];<0.0001***
Protein, g/L	0.95[0.95-0.96];<0.0001***	0.96[0.94-0.97];<0.0001***	0.98[0.96-1.01];0.2440	0.90[0.89-0.91];<0.0001***	0.96[0.94-0.98];0.0001***	0.86[0.85-0.87];<0.0001***
Creatinine, umol/L	1.002[1.002-1.002];<0.0001***	1.002[1.002-1.003];<0.0001***	1.002[1.002-1.003];<0.0001***	1.002[1.002-1.002];<0.0001***	1.003[1.002-1.003];<0.0001***	0.98[0.98-0.99];<0.0001***
Alkaline phosphatase, U/L	1.004[1.003-1.004];<0.0001***	1.003[1.003-1.004];<0.0001***	1.00[1.00-1.01];<0.0001***	1.006[1.006-1.007];<0.0001***	1.01[1.00-1.01];<0.0001***	1.006[1.006-1.007];<0.0001***
Aspartate transaminase, U/L	1.000[1.000-1.001];0.0680	0.999[0.995-1.003];0.7131	1.001[0.999-1.002];0.3027	0.96[0.96-0.97];<0.0001***	1.00[0.99-1.01];0.6976	0.89[0.88-0.89];<0.0001***
Alanine transaminase, U/L	0.99[0.98-0.99];<0.0001***	0.99[0.98-1.00];0.0007***	1.00[0.99-1.01];0.7829	0.98[0.97-0.98];<0.0001***	0.99[0.98-0.99];0.0014**	0.97[0.96-0.97];<0.0001***

Bilirubin, umol/L	1.00[0.99-1.00];0.2121	0.99[0.97-1.00];0.1122	1.01[0.99-1.02];0.3265	1.02[1.01-1.02];<0.0001***	1.00[0.98-1.02];0.7078	1.02[1.02-1.03];<0.0001***
Glycemic and lipid profiles						
Triglyceride, mmol/L	0.94[0.92-0.97];<0.0001***	0.98[0.92-1.04];0.4678	1.05[0.97-1.15];0.2191	1.05[1.03-1.07];<0.0001***	0.96[0.88-1.05];0.4048	1.12[1.10-1.14];<0.0001***
Total cholesterol, mmol/L	0.99[0.96-1.01];0.2148	1.03[0.97-1.09];0.3278	1.03[0.92-1.16];0.6300	1.22[1.19-1.25];<0.0001***	1.04[0.95-1.14];0.4010	1.60[1.54-1.65];<0.0001***
Low-density lipoprotein (LDL), mmol/L	0.95[0.91-0.99];0.0131*	0.98[0.88-1.08];0.6639	0.86[0.70-1.07];0.1782	1.21[1.16-1.26];<0.0001***	0.91[0.78-1.06];0.2358	1.61[1.52-1.70];<0.0001***
High-density lipoprotein (LDL), mmol/L	0.91[0.83-1.00];0.0609	0.55[0.42-0.72];<0.0001***	0.77[0.47-1.27];0.3016	1.01[0.90-1.14];0.8532	0.51[0.33-0.78];0.0019**	0.81[0.67-0.99];0.0360*
Fast glucose, mmol/L	1.02[1.01-1.02];<0.0001***	1.02[1.00-1.04];0.0140*	1.04[1.01-1.07];0.0051**	1.05[1.04-1.05];<0.0001***	1.04[1.01-1.06];0.0008***	1.07[1.06-1.08];<0.0001***
HbA1C, g/dL	0.97[0.96-0.97];<0.0001***	0.96[0.95-0.97];<0.0001***	0.96[0.94-0.99];0.0119*	1.01[1.00-1.01];0.1101	0.95[0.93-0.97];<0.0001***	1.09[1.07-1.11];<0.0001***

Supplementary Table 5. Sensitivity analysis 2: hazard ratios for associations of SGLT2I v.s. DPP4I using Cox proportional hazard model for adverse cognitive dysfunction and mortality outcomes in matched cohorts, with 1-year lag time.

* for p≤ 0.05, ** for p ≤ 0.01, *** for p ≤ 0.001; SGLT2I: Sodium-glucose cotransporter-2 inhibitors; DPP4I: Dipeptidyl peptidase-4 inhibitors; HR: hazard ratio; CI: confidence interval.

**Adverse outcomes**	**SGLT2I v.s. DPP4I (After 1:2 matching) HR [95% CI];P value**
New onset Parkinson’s	0.29[0.11-0.92];0.0112*
New onset Alzheimer’s	0.24[0.05-1.02];0.0511.
New onset dementia	0.42[0.34-0.73];<0.0001***
Cerebrovascular mortality	0.37[0.24-0.57];<0.0001***
Cardiovascular mortality	0.65[0.42-0.82];<0.0001***
All-cause mortality	0.77[0.68-0.89];<0.0001***

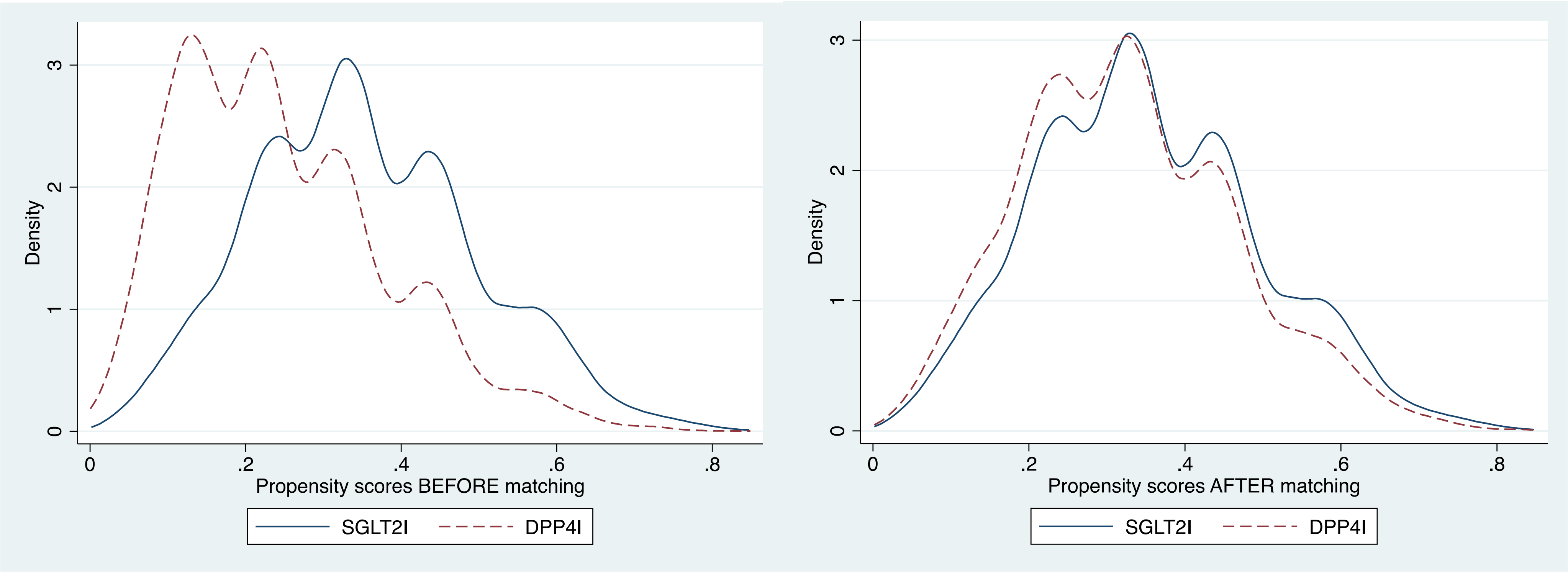

Supplementary Figure 1. Propensity score matching for SGLT2I use versus DPP4I use before and after 1:2 matching with nearest neighbor search strategy.
